# Endometabolic profiling of pigmented glacier ice algae: the impact of sample processing

**DOI:** 10.1007/s11306-024-02147-6

**Published:** 2024-08-09

**Authors:** Elisa K. Peter, Carsten Jaeger, Jan Lisec, R. Sven Peters, Rey Mourot, Pamela E. Rossel, Martyn Tranter, Alexandre M. Anesio, Liane G. Benning

**Affiliations:** 1 German Research Centre for Geosciences – GFZ, 14473 Potsdam, Germany; 2 Department of Earth Sciences, Freie Universität Berlin, 12249 Berlin, Germany; 3 Bundesanstalt für Materialforschung und -prüfung (BAM), 12489 Berlin, Germany; 4 Department of Environmental Science, Aarhus University, 4000 Roskilde, Denmark

**Keywords:** Metabolomics, High resolution mass spectrometry, Glacier ice algae, Greenland, FlowCam, PAM fluorometry

## Abstract

**Introduction:**

Glacier ice algae, mainly *Ancylonema alaskanum* and *Ancylonema nordenskiöldi,* bloom on Greenland Ice Sheet bare ice surfaces. They significantly decrease surface albedo due to their purple-brown pigmentation, thus increasing melt. Little is known about their metabolic adaptation and factors controlling algal growth dynamics and pigment formation. A challenge in obtaining such data is the necessity of melting samples, which delays preservation and introduces bias to metabolomic analysis. There is a need to evaluate the physiological response of algae to melting and establish consistent sample processing strategies for metabolomics of ice microbial communities.

**Objectives:**

To address the impact of sample melting procedure on metabolic characterization and establish a processing and analytical workflow for endometabolic profiling of glacier ice algae.

**Methods:**

We employed untargeted, high-resolution mass spectrometry and tested the effect of sample melt temperature (10, 15, 20 °C) and processing delay (up to 49 h) on the metabolome and lipidome, and complemented this approach with cell counts (FlowCam), photophysiological analysis (PAM) and diversity characterization.

**Results and Conclusion:**

We putatively identified 804 metabolites, with glycerolipids, glycerophospholipids and fatty acyls being the most prominent superclasses (> 50% of identified metabolites). Among the polar metabolome, carbohydrates and amino acid-derivatives were the most abundant. We show that 8% of the metabolome is affected by melt duration, with a pronounced decrease in betaine membrane lipids and pigment precursors, and an increase in phospholipids. Controlled fast melting at 10 °C resulted in the highest consistency, and is our recommendation for future supraglacial metabolomics studies.

**Supplementary Information:**

The online version contains supplementary material available at 10.1007/s11306-024-02147-6.

## Introduction

The Greenland Ice Sheet (GrIS) is the second largest body of ice on Earth and at present the largest single cryospheric contributor to sea level rise. There has been a significant increase in annual ice mass loss during the past decades, with increased surface melt being the main cause (Mouginot et al., 2019; Shepherd et al., 2020; Van den Broeke et al., 2017). This increased melt is due to an extension of the melting surface area, as well as a significant reduction of the reflectivity, or albedo, of the ice surface (Box et al., 2012; Enderlin et al., 2014; Feng et al., 2023). Albedo reduction, a distinct darkening of the surface, results in higher radiative energy transfer from solar radiation to the ice, thus accelerating melt (Feng et al., 2023; Tedstone et al., 2020).

Surface darkening is linked to both pigmented red and green snow algae and purple-brown glacier ice algae, which are part of the characteristic microbial communities inhabiting the surface of glaciers and ice sheets (Anesio et al., 2017; Chevrollier et al., 2022; Cook et al., 2020; Hotaling et al., 2021; Williamson et al., 2020; Yallop et al., 2012). Snow microalgal communities are dominated by *Chlamydomonas, Chloromonas* and *Sanguina* species (Lutz et al., 2016; Procházková et al., 2019; Remias et al., 2005), while on bare ice, microbial communities are dominated by the Zygnematophycean microalgae *Ancylonema alaskanum* and *Ancylonema nordenskiöldi* (hereafter glacier ice algae, GIA) (Anesio et al., 2017; Di Mauro et al., 2020; Remias et al., 2012a, 2012b; Winkel et al., 2022). A first analysis of albedo reduction by GIA on the GrIS estimated that high algal biomass patches increased melt by between 13% and up to 26% (Cook et al., 2020). The water-soluble phenolic pigment, purpurogallin carboxylic acid-6-*O-β*-d-glucopyranoside, hereafter purpurogallin, is responsible for the dark coloration of GIA, and is found to accumulate in large amounts in vacuoles surrounding the nucleus and chloroplasts of GIA (Remias et al., 2009a, 2009b, 2012a, 2012b). The production of purpurogallin is assumed to be a physiological adaptation, allowing GIA to thrive on glacier surfaces, by (1) shielding their DNA and vital organelles from damage by excessive solar radiation and (2) increasing their heat uptake on the surrounding ice, allowing a film of liquid water to be maintained (Remias et al., 2009a, 2009b; Williamson et al., 2020). Understanding the role and composition of typical GIA pigments including purpurogallin as well as photosynthetic pigments and photoprotective carotenoids (Halbach et al., 2022; Remias et al., 2012a, 2012b; Williamson et al., 2020), has allowed a simplified incorporation of the snow and glacier ice algae biological albedo reduction into predictive melt models (Chevrollier et al., 2022; Cook et al., 2020; Onuma et al., 2020). However, a far improved quantification of the physical and biogeochemical parameters controlling GIA growth dynamics and pigment production is needed to refine melt models and more accurately predict biological albedo effects in a warming climate.

Untargeted metabolomics is a promising tool from which to obtain relevant data on GIA adaptations, growth dynamics, pigment formation and microbial interaction. However, to date only very few studies addressed supraglacial microbial metabolomics (Davey et al., 2019; Gokul et al., 2023; Lutz et al., 2015; Procházková et al., 2019, 2021). They all use different sampling and sample processing strategies and thus it is difficult to impossible to achieve comparability between studies. In supraglacial environments, for example, chlorophyte snow algae community metabolomes that are rich in carotenoids have been studied from Svalbard, Norway (Lutz et al., 2015; Procházková et al., 2019) and Antarctica (Davey et al., 2019). In addition, only one study on total metabolites in GIA dominated surface ice microbial communities (Lutz et al., 2024), one on exometabolites of GIA dominated surface ice (Doting et al., 2024) from the Greenland Ice Sheet and one study on the lipidomics of GIA rich samples from the Swiss and Austrian Alps (Procházková et al., 2021) have been published to date. Furthermore, a few targeted pigment analyses of snow (Lutz et al., 2015; Remias et al., 2005) and glacier ice algae rich samples (e.g., Chevrollier et al., 2022; Halbach et al., 2022; Williamson et al., 2020) are available. However, differences in sample handling following collection, including for example variable processing delays (up to 24 h and more) due to the need to melt the surface ice sample at variable—often unspecified—low ambient temperatures (0–10 °C) and variable light conditions are aspects in the methodologies in many of these studies that will invariably impact metabolite compositions.

The delay in processing stems from the fact that surface snow and ice samples are essentially frozen suspensions, with particulate material (algae, other microorganisms, and mineral components) held within a frozen snow or ice matrix. Accordingly, samples must either be quench-melted in whole through direct solvent addition to the frozen sample, thus mixing the exo- and endometabolome, or the snow or ice sample must first be melted to allow separation of particulates prior to quenching and preservation. Direct quenching of the sample is undesirable in most cases, as it (1) dilutes the endometabolome with the extracellular snow or ice matrix, potentially below the limit of detection for sparse metabolites, (2) makes it impossible to separately analyze the endo- and exometabolome, (3) prevents sub-sampling for additional analyses from the same sample, and (4) requires large solvent volumes especially when sampling low-biomass snow and ice, which is additionally logistically challenging in remote locations. However, if samples are melted prior to quenching, long melt durations allow algae to adapt to the changing environmental conditions upon removal from their natural habitat, so that melted and processed samples may not accurately reflect the true metabolome at the time of sampling (Alseekh et al., 2021; Lu et al., 2017). Processing delays and the unknown effects that arise from the need to melt the sample, are of concern for many geochemical (e.g., dissolved and particulate carbon or other nutrient signals), proteomic and transcriptomic analyses. However, they are of particular concern for metabolomics, as metabolic degradation and turnover rates can be of the order of seconds (Lu et al., 2017; Mashego et al., 2007; van Gulik, 2010). Ultimately, the true metabolome of GIA in their natural habitat is impossible to ascertain. However, we can determine the impact of various sample processing strategies and establish standard methods to limit variability and enable comparisons between studies.

We tested the effect of melt duration and processing delay at ambient conditions, as well as melt temperature in a water bath, on the metabolome of GIA dominated microbial communities from GrIS surface ice, in order to minimize potential methodological artefacts in the metabolome of GIA in future studies. We further report the first untargeted endometabolic characterization of this environment.

## Methods and materials

### Site description, sampling and sample handling

**Fig. 1:**
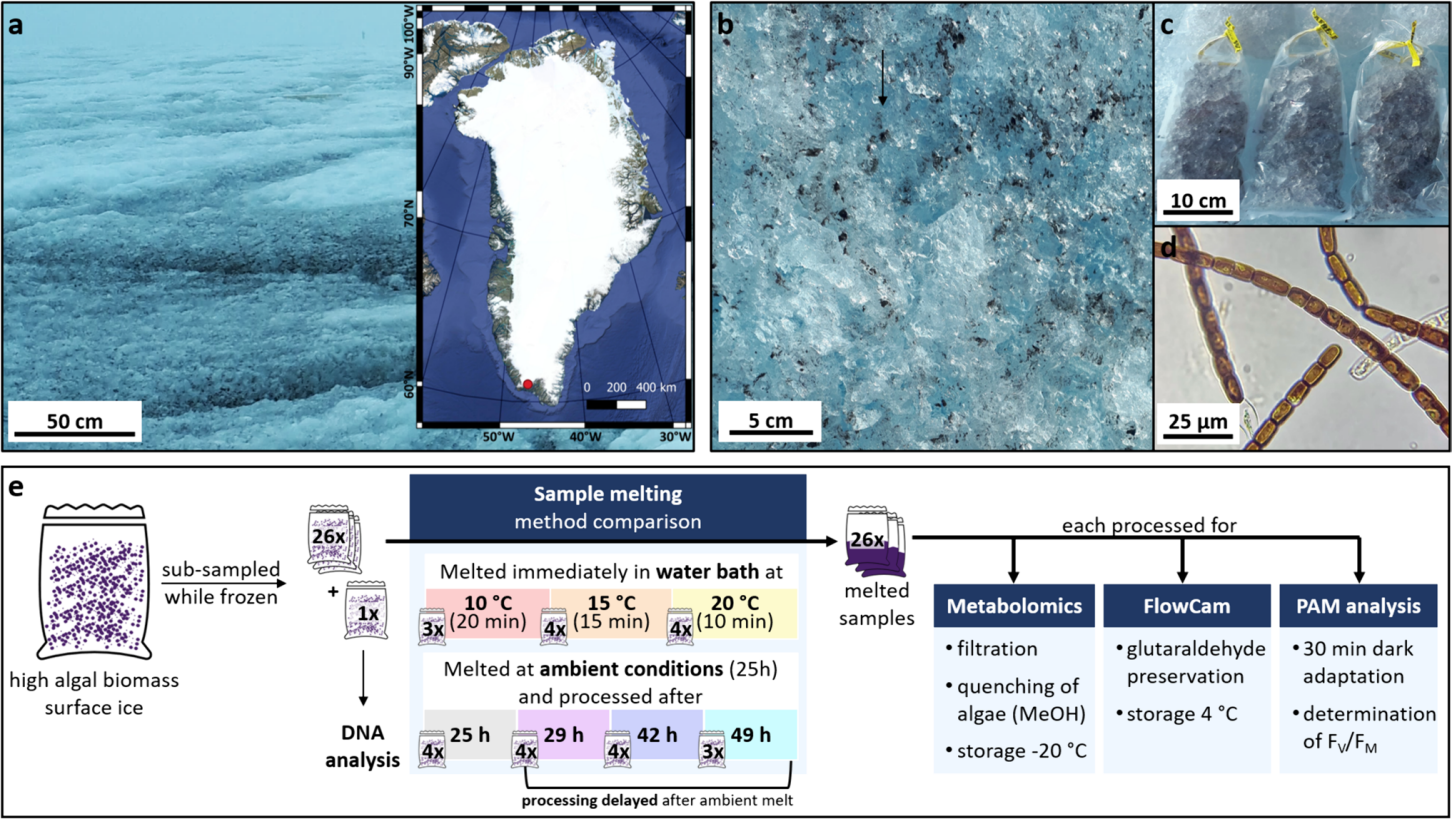
**a** Sampling site located at the 2021 DEEP PURPLE field site on the southern Greenland Ice Sheet margin at 61°05’N, 46°50’W, (**b, c, d**) representative detail images of the sampled surface ice, sub-divided ice samples in sample bags before melting and a light microscopy image (100 fold magnification, VisiScope100, Model BL124) of glacier ice algae dominated microbial communities directly after sample melting. **e** Sample processing workflow showing the treatment of sub-samples to assess the impact of melt temperature (WB samples), melt duration and delayed processing (AM samples) on the meta-metabolome of surface ice samples that are rich in algal biomass

Sampling was conducted on August 2nd 2021 at 2.30 PM on the southern GrIS margin, at an elevation of 623 m at 61°05’N, 46°50’W, upwind of the DEEP PURPLE (deeppurple-ercsyg.eu) base camp (Fig. 1a). The sky was overcast, with rain throughout the previous day and until noon of the sampling day. Average daily air temperatures were downloaded from the PROMICE automatic weather station QAS-M (Fausto et al., 2021), located approximately 900 m east of the sampling location. Sampling occurred around the peak light intensity of the day. The maximum downwelling shortwave radiation was recorded 1.5 h prior to sampling at 35.9 W/m^2^ (Fausto et al., 2021). The top 2 cm of the surface ice from a 50 × 50 m area containing patches of visibly high concentrations of algal biomass were collected into a sterile 8 l sample bag (Whirl–Pak), resulting in 4.2 kg of ice. Sampling was undertaken with an on-the-ice pre-conditioned ice axe and metal trowel.

This frozen bulk sample was homogenized within the large collection bag and subsequently split into 27 sterile 450 mL sample bags (Whirl–Pak) to create replicate samples (Fig. 1e). The total time from the beginning of sample collection to obtaining 27 sub-samples was 1.5 h. One of the bags was processed for DNA analysis, while the remaining 26 sub-sampled bags were divided into seven groups of 3–4 replicates each.

The first three sample groups were melted in temperature-controlled water baths at 10, 15 and 20 °C (WB samples, hereafter labeled WB10C, WB15C and WB20C respectively) which took 20, 15 and 10 min respectively to melt (Fig. 1e). Immediately after a group of sample bags were melted, 45 ml of each homogenized sample was filtered through a pre-combusted glass fiber filter (25 mm, 0.7 µm pore size, Whatman) using a vacuum filtration setup. Filters with particulates were transferred into 2 ml microcentrifuge tubes and quenched with 50% methanol (MeOH) in water (both LC–MS grade, Fisher chemicals). Filters and sample tubes were weighed prior to fieldwork so that sample dry weight could be determined after freeze drying (see Sect. 3.2). The remaining four sample groups were left to melt at the local ambient temperature, in a shaded area of the field laboratory tent. The first group of replicates was processed as above, immediately after they were completely melted, which was after 25 h (AM25h). Filtering and quenching were delayed for the other three groups of sub-samples for an additional 4, 17 and 24 h after they were fully melted (after 25 h; hereafter labeled AM29h, AM42h and AM49h respectively). Following filtration and quenching, all samples were stored at − 20 °C until the end of the field season (August 20th 2021) and transported at − 20 °C to Potsdam, Germany where they arrived on September 22nd 2021. Until further processing (see Sect. 3.2), samples were then kept at − 80 °C.

A 20 mL aliquot from each of the 27 sub-sampled bags was reserved for pulse amplitude modulated fluorometry (PAM) analysis while on the ice, and a further 10 mL aliquot was fixed with glutaraldehyde (final concentration of 2.5%) and preserved at 4 °C for cell count analysis to be performed in Potsdam (see Sect. 3.3). A melted sub-sample was also directly imaged on site using light microscopy (100-fold magnification, VisiScope100, BL124) (see Fig. 1d).

### Metabolomics sample extraction, analyses and data processing

Samples were vacuum concentrated (RT, 1200 rpm, 8 mbar, RVC 2–25 CDplus, Christ) to approximately 50% of the original sample volume to remove methanol, freeze-dried (Scanvac CoolSafe, Labogene) and stored at − 80 °C until extraction. Samples were weighed prior to extraction. The average extracted dry weight per sample was 37 mg (for individual sample weights see S1, Table 1). A sequential extraction protocol based on the biphasic solvent extraction by Sostare et al. (2018) was developed for improved metabolite recovery. Two stainless steel milling balls (3 mm, Retsch) and 1 ml of the extraction solvent (Methyl *tert*-butyl ether (MTBE):MeOH, 3:1 *v/v* with internal standard mix Cambridge Isotope labs MSK-QC MIX dissolved in 50% MeOH and 0.1% w/v BHT, − 15 °C cold) were added to each 2 ml freeze-dried sample tube. The tubes were flash-frozen in liquid nitrogen for 10 s and milled (2 min, 30 Hz, MM400, Retsch, sample holder pre-cooled at − 20 °C). A total of 3 flash-freeze and milling cycles were employed, with the last milling extended to 10 min. Milled samples were ultrasonicated (15 min, ice-cooled, S40 H, Elmasonic), 500 µl of 25% MeOH was added, and samples were extracted, while milling (2 min, 30 Hz, MM400, Retsch). This was followed by an incubation on ice for 10 min, and centrifugation (5 min, 14,500 rpm, miniSpin plus, Eppendorf) to assist phase separation. Subsequently, 700 µl of the upper phase was transferred to a microcentrifuge tube. To increase recovery, samples were re-extracted twice with 500 µl MTBE, (milling during extraction and centrifugation as above) and each time 600 µl of the upper phase was transferred to the same tube, pooling all three extracts. Next, 300 µl of the lower, polar phase was transferred to a 15 ml centrifuge tube. The cell debris was then re-extracted, once with 800 µl 25% MeOH and then three times with 800 µl water. The samples were vortexed after each solvent addition, incubated for 10 min and 800 µl of the resulting extract collected after centrifugation. All polar extracts were pooled. Polar (MeOH–water) and non-polar (MTBE) extracts were centrifuged to remove carried-over debris. A 1 ml aliquot of the polar extract and a 600 µl of the non-polar extract were dried (30 °C, 1200 rpm, 8 mbar, RVC 2–25 CDplus, Christ), and stored under N_2_ at − 80 °C until analysis via gas chromatography-mass spectrometry (GC–MS, MeOH–water extract) and liquid chromatography-mass spectrometry (LC–MS, MTBE extract) respectively. Further information on metabolomics sample extraction is provided in Supplementary material S1.

Lipidomics LC–MS analysis was performed on a 1290 ultra-high-performance LC (UHPLC) system (Agilent, Germany), coupled to a TripleTOF 6600 high-resolution quadrupole time-of-flight mass spectrometer (Sciex, Germany). Dried sample extracts were reconstituted in 100 μl 60:30:4.5* v/v/v* chloroform:methanol:water. 5 μl aliquots were injected using a randomized sample sequence. Chromatographic separation was achieved by gradient elution (%A: 0 min, 60; 1.2 min, 57; 1.26 min, 50; 7.2 min, 46; 7.26 min, 30; 10.8 min, 0; 12.96 min, 0; 13.02 min, 60; 14.4 min, 60) using a binary solvent system (A: 60:40 v/v acetonitrile/water, B: 90:10 v/v isopropanol:water, both with 10 mM ammonium formate and 0.1% formic acid) on a 2.1 mm × 75 mm × 1.7 μm CSH-C18 column (Waters, Germany), which was held at a temperature of 55 °C. The flow rate was 0.5 mL min^−1^. Electrospray ionization (ESI) was carried out in positive (+) and negative (−) mode using capillary voltages of 5500 and 4500 V, respectively. The ion source temperature was 450 °C. Full scan MS data was acquired in the *m/z* range of 100–1250. MS/MS spectra were recorded at 35 eV (spread 20 eV) in data-dependent mode, auto-selecting the four highest *m/z* features per peak (DDA Top-4 mode). The cycle time was 0.8 s. Mass calibration was performed at the beginning of the sequence using an ESI( +) or ESI( −) tune mix, respectively. For data analysis, MS files were converted to centroid mzML format and imported into MS-DIAL (Tsugawa et al., 2015). MS-DIAL parameter settings were as follows: Soft Ionization, Data dependent MS/MS, Centroid data, Positive ion mode, Lipidomics. Detailed analysis settings were left at default, except for retention time end (10 min), alignment retention time tolerance (0.2 min), identification retention time tolerance (3 min) and identification score cut off (60%).

The polar metabolome was analysed via GC–MS following trimethylsilyl derivatization. Samples were reconstituted in 80 µl methoxyamine solution (20 mg/ml in pyridine, freshly prepared) and incubated for 90 min at 30 °C on an orbital shaker (Eppendorf Thermoshaker; 1200 rpm). 160 µl MSTFA (*N*-methyl-*N*-trimethylsilyl-trifluoroacetamide; Macherey–Nagel; order no. 701270.201) were added after quick centrifugation (1 min, 14,500 rpm, miniSpin plus, Eppendorf) and samples were incubated for 30 min at 37 °C on an orbital shaker. 1 µl derivatized sample was injected into an 8890 GC (Agilent, Germany), coupled to a 7250 high-resolution quadrupole time-of-flight mass spectrometer (Agilent, Germany). Injection was performed at 280 °C in pulsed splitless mode. Separation was performed on a HP-5MS Ultra Inert column (30 m × 0.25 mm × 0.25 µm; Agilent, Germany) using Helium as a carrier gas at a flow rate of 1.1 ml min^−1^ and a temperature gradient from 80 to 320 °C at an increase rate of 15 °C min^−1^. The total runtime was 23 min. Mass spectrometric detection was performed using electron ionization (70 eV), a mass range of 40–600 Da and a scan rate of 4 s^−1^. For data analysis, MS files were converted to centroid mzML format and imported into MS-DIAL (Tsugawa et al., 2015). MS-DIAL parameter settings were as follows: hard ionization, conventional LC/MS or data dependent MS/MS, centroid data, positive ion mode, metabolomics. The Golm Metabolite Database (GMD) was used as the reference spectral library (Kopka et al., 2005). Purpurogallin and its glycoside derivatives are not amenable to GC–MS and are therefore not included in our analysis.

Raw data was pre-processed in MS-DIAL and annotated LC–MS and GC–MS feature tables, were imported into an R workspace (R Core Team, 2021). To normalize variance in feature intensity based on technical factors, median peak intensity per sample, extracted sample dry weight and run order in measurement batch, were removed for each metabolic feature using an ANOVA based approach (Jaeger & Lisec, 2018). Further statistical and multivariate analyses were performed using MetaboAnalyst 5.0 (Pang et al., 2021), after log transformation and mean centring of intensities.

### Pulse amplitude modulated fluorometry (PAM) and cell counts (FlowCam)

The fluorescence ratio, *F*_*V*_*/F*_*M*_, was determined for all samples as an estimate of the maximum photochemical quantum yield of photosystem II using a WaterPAM fluorometer (Walz GmBH). Aliquots of the 26 melted sample bags were dark adapted for 30 min prior to analysis. Blank adjustment was done for each group of samples by filtering a replicate sample through a 0.2 µm syringe filter. The minimum and maximum fluorescence levels, *F*_*0*_ and *F*_*M*_, were determined and *F*_*V*_*/F*_*M*_ ratios then calculated (Kitajima & Butler, 1975), where *F*_*V*_ = *F*_*M*_ − *F*_*0*_. A one-way ANOVA and post-hoc Tukey test were performed in RStudio to compare the average *F*_*V*_*/F*_*M*_ ratios between melt treatments.

Cell numbers were determined using a FlowCam 5000 particle analyser (Yokowaga Fluid Imaging Technologies) equipped with a 10 × objective, using the associated Visual Spreadsheet 5 software. Particles were classified as filamentous and unicellular GIA, as well as snow algae (see supplementary text and supplementary Fig. 1). We used the approach of Williamson et al. (2018) for cell volume calculation of GIA based on a cylindrical model (Hillebrand et al., 1999), using cell diameter and cell height measurements obtained in ImageJ on GIA FlowCam images. Carbon content was then calculated using the cell volume to carbon content ratio of Montagnes et al. (1994). Further details of cell classification and biovolume evaluation are provided in Supplementary information S1.

### Microbial diversity

We also analysed the microbial diversity in our bulk sample. For this, 200 ml of the ambient melted sample was filtered through a single use, sterile, 0.2 µm cellulose nitrate filter (Thermo Scientific Nalgene), transferred into a sterile cryotube, flash-frozen and transported to the home laboratory in a cryo-shipper (CX-100, Worthington Industries, Burscheid, Germany). DNA was extracted using the PowerSoil Pro kit (QIAgen) according to the manufacturer’s protocol. We used the prokaryotic primers for the 16S rRNA gene Bakt_341F (CCTACGGGNGGCWGCAG) and Bakt_805R (GACTACHVGGGTATCTAATCC) (Herlemann et al. 2011), eukaryotic primers for the 18S rRNA gene using 528F (5’-GCGGTAATTCCAGCTCCAA-3 ‘) and 706R (5’-AATCCRAGAATTTCACCTCT-3 ‘) (Cheung et al. 2010), and the internal transcribed spacer 2 (ITS2 snow) gene primers 5.8SbF (5′-GATGAAGAACGCAGCG-3′; (Mikhailyuk et al. 2008)) and ITS4R (5′-TCCTCCGCTTATTGATATGC-3′; (White et al. 1990)) and the ITS2 ice primers 5.8SbF (5’-CGATGAAGAACGCAGCG-3’) and LSULP (5’-AATTCGGCGGGTGGTCTTG-3’) (Remias et al., 2023) for PCR amplification. The final libraries were sequenced on an Illumina MiSeq using the V2 kit (Illumina Inc. SanDiego, California, US) resulting in 2 × 250 bp reads. More information on PCR, library preparation, sequencing and further data processing is available in Supplementary material S1.

## Results

### Environmental conditions, microbial diversity and cell counts

**Fig. 2:**
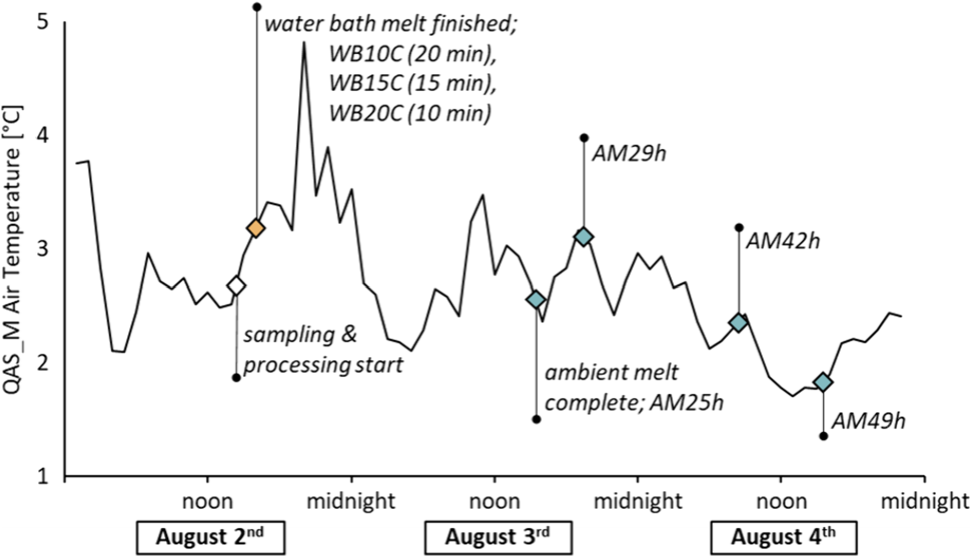
Hourly air temperature as monitored by the PROMICE automatic weather station QAS-M during August 2nd to August 4th 2021. The white diamond denotes the sampling time, the orange denotes processing in water bath (10 °C: 20 min, 15 °C: 15 min, 20 °C: 10 min) and teal diamonds denote time points in ambient melted sample processing

The ambient temperature at the sample site (measured 2 m above the ice surface at the QAS-M weather station) during the sampling and sample processing period (August 2nd to August 4th, Fig. 2) varied by approximately 3 °C (range of 1.7–4.8 °C). Processing of WB samples was completed 1.5 h after sampling. Full melt at ambient temperature was achieved after 25 h.

The 16S rRNA amplicon sequencing data revealed that the procaryotic community composition in the bulk initial sample was composed primarily of *Acidobacteria*, *Actinobacteria*, *Bacterioidota*, *Cyanobacteria*, *Deinococcota*, *Firmicutes* and *Proteobacteria* (Fig. 3 in SI), while the eukaryotic community was dominated by *Streptophytes* (*Ancylonema*, 65%), and *Chlorophytes* (*Trebouxiphycea*, *Chlorophycea*, *Chloromonas*, 15% of relative community abundance, Fig. 4 in SI)). The ITS2 ice primer showed a dominance of algal taxa such as *Hegewaldia* and members of the *Chlorophyta* phylum (Fig. 5 in SI), while *Coleophoma*, members of the *Microbotryomycetes* class and *Yamadamyces* were the main fungi taxa detected using the ITS2 snow primer (Fig. 6 in SI).

**Fig. 3:**
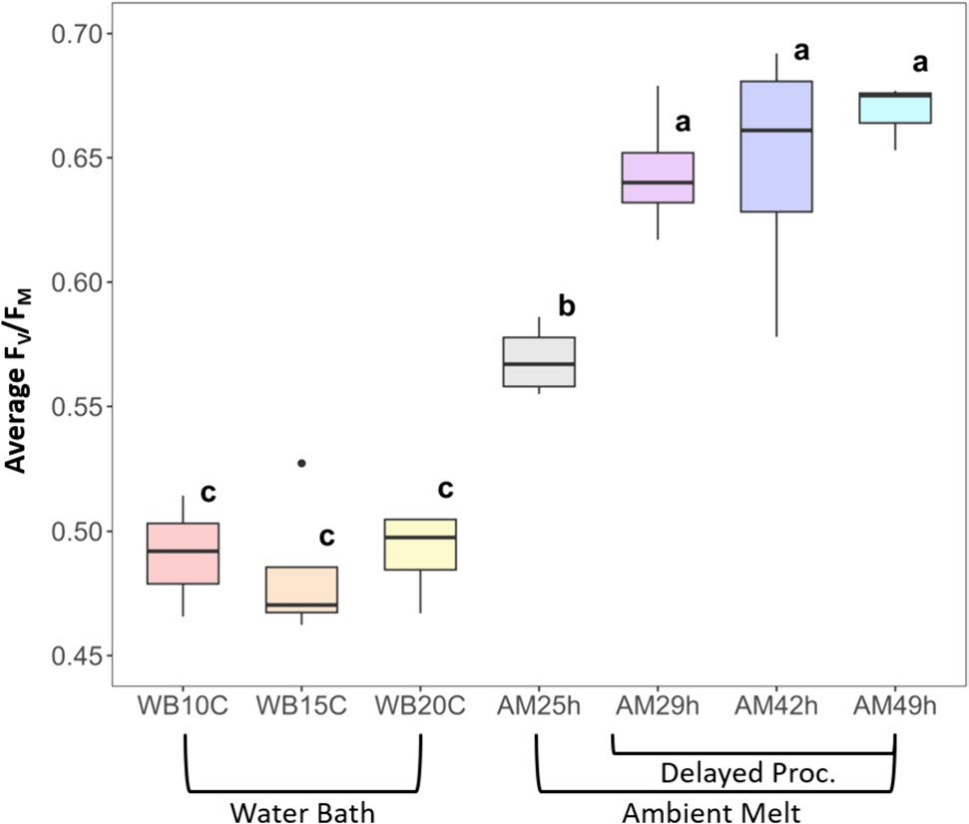
*F*_*V*_*/F*_*M*_ ratios as determined by pulse amplitude modulated fluorometry of samples melted in a water bath at 10, 15 and 20 °C, at ambient conditions and at ambient conditions with delayed processing. Bold lines in the boxes indicate the median of replicates (*n* = 3–4) within the same treatment condition. The letters on top of the boxes (**a**-**c**) denote significant differences (Tukey post-hoc, *P* < 0.05) in the pairwise comparison of *F*_*V*_*/F*_*M*_ ratio averages between treatments groups

**Fig. 4:**
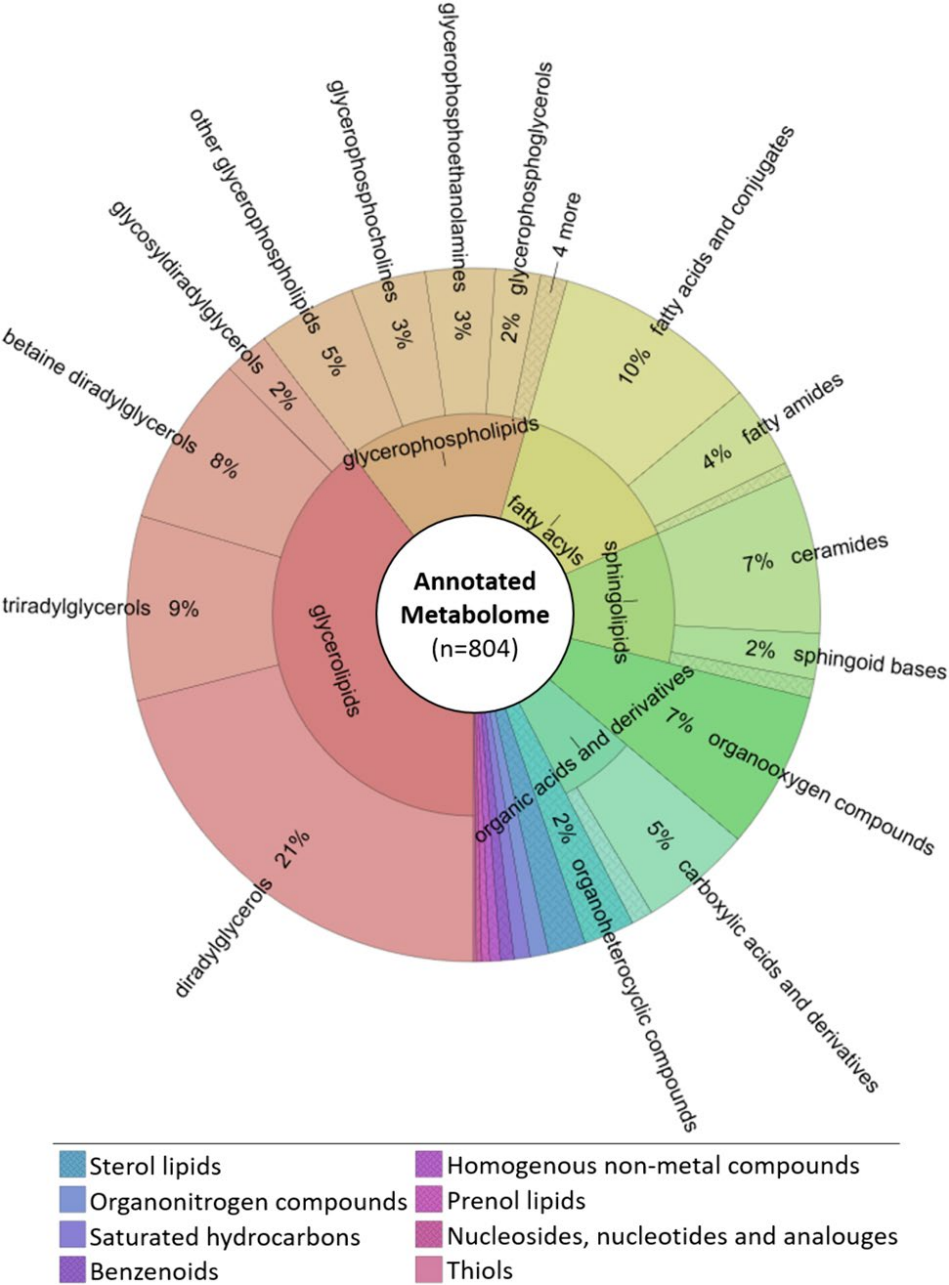
Sunburst plot illustrating range and distribution of annotated metabolites (*n* = 804) by metabolite class. The inner circle represents metabolite superclasses, the outer circle represents metabolite classes. The size of each segment corresponds to the number of metabolites found within each class. Metabolite classes with contributions of less than 2% are in the legend. The whole chart including the metabolite subclasses-layer can be interactively explored in the supplementary Html file S4

**Fig. 5:**
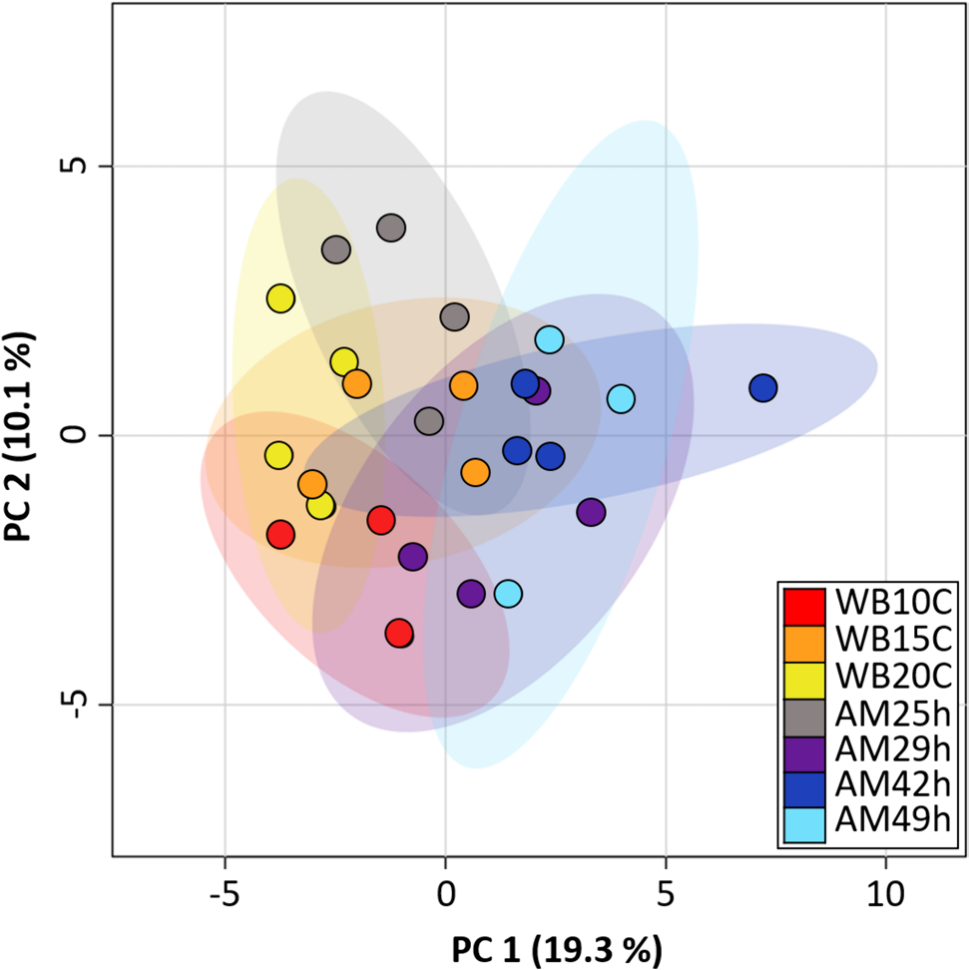
Principal component analysis (PCA) scores plot of the 7 melt treatment groups based on log10-transformed and mean centered intensities of 804 annotated metabolites. Each dot represents a sample, and color coding indicates the experimental group. Ellipses display the 95% confidence interval

**Fig. 6:**
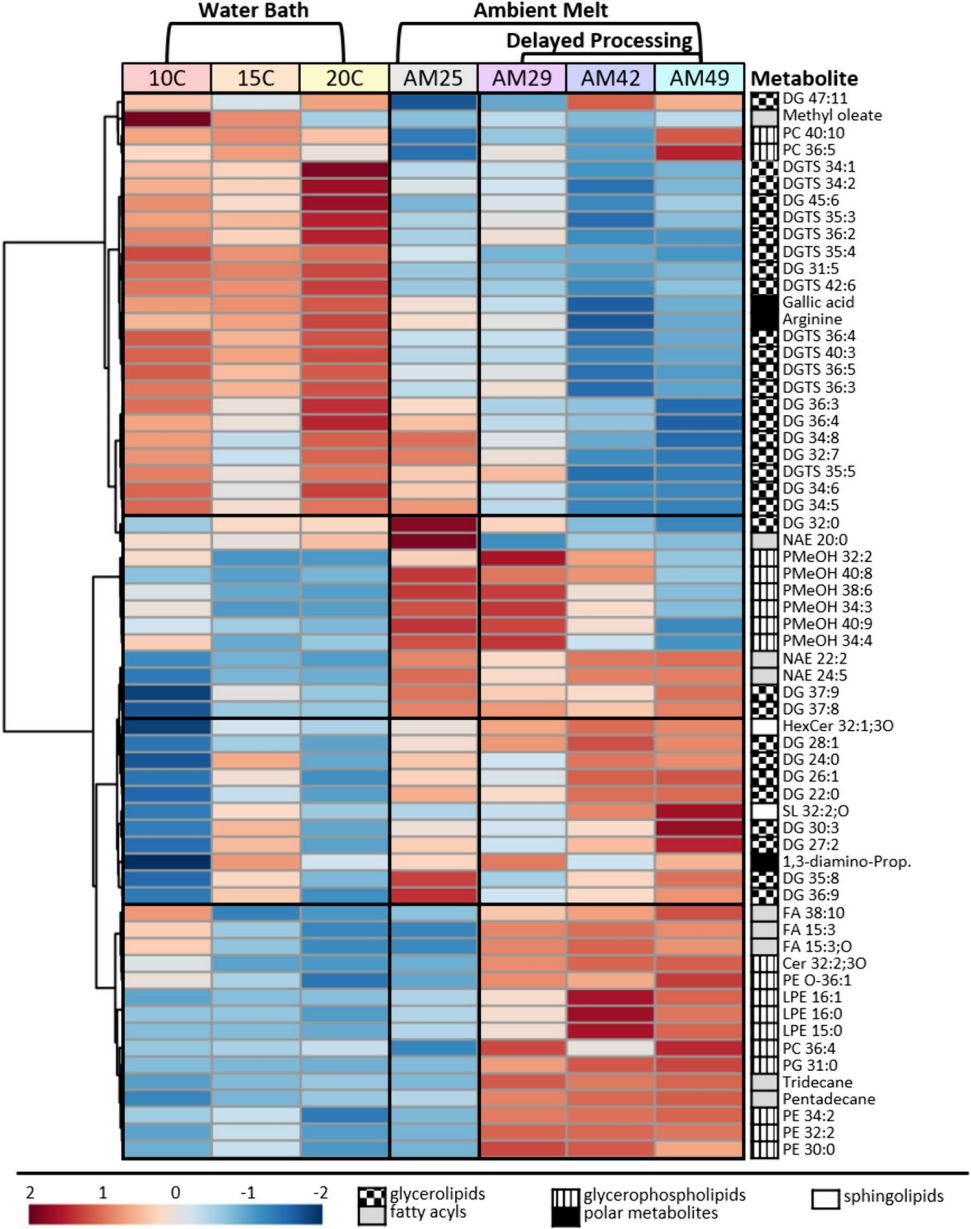
Heatmap of 63 metabolites that showed significant differences in group averages between melt treatments based on a one-way ANOVA (*p* < 0.05, FDR corrected). The color scale indicates normalized and autoscaled intensity values, with red representing high intensity and blue representing low intensity. Metabolites are clustered hierarchically using Euclidean distance and the Ward clustering algorithm. Lipid superclasses are denoted with patterned boxes. Non-lipid metabolites were summarized in one group

Using FlowCam analysis an average of 77,000 particles were imaged per sample of which approximately 4% were classified as filamentous GIA, unicellular GIA or snow algae. Most GIA appeared as filaments (see Fig. 1 in S1) and each filament contained between two to more than 40 cells per filament. On average, each filament contained eight cells. The average number of individual GIA cells linked in filaments across all 26 sub-samples was 37,987 ± 9981 cells/ml. Individual unicellular GIA cells (51 ± 19 cells/ml) and snow algae cells (133 ± 61 cells/ml) were far fewer, and made up less than 0.4% of the total number of cells/ml in a sample. The biovolume derived for individual GIA cells was 2130 ± 202 µm^3^. This biovolume translates to an average algal biomass of 8 ± 0.2 µgC/ml. Full FlowCam results are provided in S1.

### Pulse amplitude modulated fluorometry

Overall, we documented (Fig. 3) an increase in *F*_*V*_*/F*_*M*_ and photosynthetic efficiency with increasing melt duration under shaded light conditions in PAM analysis. Three clusters of significantly different (Tukey post-hoc, *P* < 0.05) *F*_*V*_*/F*_*M*_ ratios were found between the seven sample group treatments. The lowest *F*_*V*_*/F*_*M*_ ratios, and therefore highest stress levels, were found in WB samples (average 0.49 ± 0.01). Melting the samples fast and at different temperatures (WB data groups) revealed no significant difference between samples (10, 15 and 20 °C). By comparison, samples that were melted at ambient condition and for longer durations, showed a lower stress level with an *F*_*V*_*/F*_*M*_ ratio of 0.57 ± 0.01 in AM25h, and AM29h to AM49h showed the lowest stress levels at an average *F*_*V*_*/F*_*M*_ ratio of 0.66 ± 0.03. There was no significant difference in the *F*_*V*_*/F*_*M*_ ratios of ambient melted samples with processing delay durations (AM29h–AM49h).

### Metabolome of the glacier microbial community

Across all samples a common set of 2519 unique features were detected by GC–MS analysis, and 3065 unique features were detected by LC–MS/MS in positive and negative ion mode combined. Out of those, 178 GC-metabolites (7%) and 626 LC-metabolites (20%) received a level 2 annotation (Schrimpe-Rutledge et al., 2016) based on retention time and reference mass spectra.

A sunburst plot created using Krona (Ondov et al., 2011, Fig. 4) enables an assessment of the diversity in compound classes represented in our analysis, and shows that lipids and lipid-like molecules were the most commonly detected metabolites. Lipids made up the majority of the annotated metabolome, with glycerolipids dominating (40% of the total metabolome, mainly diradylglycerols, triradylglycerols, betaine diradylglycerols), followed by glycerophospholipids (15%, mainly phosphatidylmethanols, glycerophosphocholines, glycerophosphoethanolamines), fatty acyls (14%, mostly fatty acids and conjugates and fatty amides) and sphingolipids (10%, ceramides, sphingoid bases and neutral glycosphingolipids). Sterol lipids (2%, mainly cholesterols and sterol sulfates) and prenol lipids (0.4%) were minor lipid components. Organooxygen compounds (7% of metabolome, mainly carbohydrates and conjugates) and organic acids (6% of the metabolome, mainly amino acids) were dominant among the polar metabolome. Additional, yet minor (2% or less of the total metabolome), polar metabolite classes included organoheterocyclic compounds, organonitrogen compounds, saturated hydrocarbons, benzenoids, homogenous non-metal compounds, nucleosides and analogues, and alkylthiols.

### Effects of melt temperature and processing duration on the metabolome

We assessed the impact of melt treatments on the overall metabolome (*n* = 804), via principal component analysis using log10-transformed, mean centered intensity values of annotated metabolites (PCA, Fig. 5). The first two principal components accounted for 19.3 and 10.1% of the total variation. The analysis shows low variability among both the water bath treated (WB10–20C) and the ambient melt sample groups with delayed processing (AM29–49 h) which separate along PC1. The AM25h samples showed the clearest distinction, grouping in between water bath melted samples and ambient melt with delayed processing.

We performed a one-way ANOVA on each of the 804 metabolites across all sample groups, to evaluate the contribution of individual metabolites and pathways that are affected by melt treatment. We found that 63 metabolites were significantly affected (*p* < 0.05 FDR corrected), which amounts to 8% of all level 2 annotated metabolites. The heatmap in Fig. 6 reveals clear patterns of metabolite abundance across melt treatments, with lipid components most affected and many of the affected lipids showing a high degree of unsaturation. Mirroring the PCA visualization, WB and AM data sets reveal consistent, yet opposing trends for most affected metabolites. The AM25h samples, which are the transition samples between the two melt strategy groups, shared metabolome characteristics with both water bath melted samples and the ambient melted sample groups with delayed processing. Glycerolipids and glycerophospholipids are the largest affected lipid groups, with betaine (DGTS) lipids and diacylglycerides dominating in glycerolipids and phosphatidylmethanol (PMeOH), phosphatidylethanolamines (PE) and phosphatidylcholines (PC) as the largest component of affected glycerophospholipids.

DGTS lipids showed the most pronounced effect and were decreased in all AM samples, while diacylglycerides showed opposing effects, with some being increased and others decreased. PMeOH lipids were lower in water bath melted samples, increased in ambient melted samples AM25h and AM29h and decreased again after extended duration (AM42h and AM49h).

We identified six metabolites in our GC–MS data that were affected by melt treatment. Methyl oleate, gallic acid and arginine all decreased in ambient melted samples (AM25–49 h), while tridecane, pentadecane and the amino acid precursor, 1,3-diaminopropane, increased with processing duration. Gallic acid showed a particularly strong decrease with a reduction of 45% in ambient melted samples compared to water bath melt based on raw peak intensity.

## Discussion

Our objective was to make the metabolome and lipidome of GIA-rich samples accessible, by establishing an untargeted metabolomics workflow suitable for this unique, extreme environment. Simultaneously, we wanted to assess the impact of sample melting during in-field sample processing on metabolomics results and infer any possible glacier ice algae stress response. We compared water bath melting at 10, 15 and 20 °C to melting at ambient conditions for 25 h and also delayed processing after melting at ambient conditions. We were able to putatively annotate 804 metabolites (Supplementary Table S2 and S3) and found that 8% of the analysed metabolome were significantly affected by the interplay between melt duration, processing delay and temperature. Transfer to a water bath is arguably a considerable shift for GIA rich samples compared to melting slowly at ambient temperatures, yet ambient melted samples are inherently also subject to the natural variability of environmental conditions, such as light and temperature. Our data clearly shows that long melt duration and processing delay after melting are the most important factors affecting metabolome composition (Fig. 6), with a variation of increasing and decreasing trends across the affected metabolites. Combining the metabolite data with our analysis of GIA stress response using PAM fluorometry (Fig. 3) confirms the significant impact of processing conditions including light exposure, duration and temperature on GIA photophysiology.

### Local environmental conditions and microbial community composition

The ambient temperature at two-meter height during our sample processing period ranged between 1.7 and 4.8 °C (Fig. 2), which is at least 5 °C colder than the lowest water bath melt temperature (10–20 °C). Our ambient melt samples (AM29–49 h) were melted inside our field-laboratory tent, close to the tent floor. Generally, temperatures in the tent were likely higher than those measured by the QAS-M air temperature probe at 2 m height. However, the samples were near the floor of the tent and thus close to the ice surface, and so QAS-M air temperature is likely sufficient to assess ambient air temperature and variation. Full melting of the sample bags at ambient conditions was achieved after 25 h, therefore allowing much more time for physiological adaptation of the GIA compared to melting in a water bath (total of 1.5 h from sampling to quenching, including 10–20 min melt time). The large difference in melt time is due to the better heat transfer in a water bath compared to that from the surrounding air in ambient melted samples. It can be assumed that meltwater in the sample bag remained close to 0 °C during melt for both water bath and ambient melted samples, although meltwaters in the water bath are likely to be up to a few degrees warmer despite their contact with ice through most of the melting.

The microbial community composition (Supplementary Figs. 3–6) confirmed that the sample that was collected from dark surface ice contained primarily equivalent taxa, as previously described for supraglacial algal rich bare ice habitats (Anesio et al., 2017; Halbach et al., 2022; Jaarsma et al., 2023; Lutz et al., 2018; Winkel et al., 2022). The amplicon sequencing data revealed that the eukaryotic microbial community was dominated by GIA (mainly Ancylonema), yet it still contained a 15% relative contribution from snow algae (Chlorophytes; Supplementary Fig. 4). Such a relative proportion is comparable to published data describing the eukaryotic diversity in darkened bare ice environments (Halbach et al., 2022; Lutz et al., 2018; McCutcheon et al., 2021; Remias et al. 2009a, 2009b, 2012a, 2012b; Winkel et al., 2022).

To note is, that the 15% relative contribution representing chlorophytes, which typically inhabit snow surfaces (Chevrollier et al., 2022; Jaarsma et al., 2023; Lutz et al., 2015, 2017; Remias et al., 2005), was about one order of magnitude higher than the absolute snow algal cell count proportions from the FlowCam analysis (S1, Table 1) (0.35% of total cell count). The cell count for the filamentous GIA in our sample (37,987 ± 9981 cells/ml; S1, Table 1) was similar in range to previously reported values in dark ice on the SE Greenland Ice Sheet (32,000 cells/ml, Halbach et al., 2022 or 29,000 ± 20,100, Cook et al., 2020). However, we could image an average sample volume of 600 µl using the FlowCam approach for cell counts and evaluated between 480 and 4500 GIA filaments per sample, which is one to several orders of magnitude more compared to manual cell counting, where usually from 10 to 25 µl (haemocytometry chamber, Chevrollier et al., 2022; Halbach et al., 2022) up to 100 µl (Sedgewick Rafter, Chevrollier et al., 2022) or 300 cells per sample are counted (haemocytometry chamber, Cook et al., 2020; Williamson et al., 2018). The higher throughput in sample volume and cell numbers, as well as the automation of particle characterization, makes FlowCam based cell count analysis more robust.

Cell volumes determined in our analysis (2130 ± 202 µm^3^), based on cell dimension measurements in ImageJ, match with previously reported values for GIA on the SE Greenland Ice Sheet (1700–2500 µm^3^, Halbach et al.), the SW Greenland Ice Sheet (approx. µm^3^, 1300–2300 Williamson et al., 2018) and the Southern Greenland Ice Sheet (1400–1500 µm^3^, Chevrollier et al., 2022). GIA biomass (8 ± 0.2 µg C/ml) derived from our analyses, was also in the range of that reported by Halbach et al. (2022) (1.7 to 13 µg C/ml), but almost 10 times higher than that reported by Williamson et al., 2018 (approx. 1 µg C/ml). In our case, GIA biomass was made up almost entirely of filamentous algae, whereas Williamson et al. found unicellular and filamentous GIA in similar proportions. The number of unicellular GIA (not in filaments) in our study was 51 cells/ml, compared to Halbach et al. (2022) who reported that the number of *A. alaskanum* varied between 790 and 23,000 cells/ml along a snow to ice transect. This variance in unicellular to filamentous GIA likely reflects the impact of location-based variation in environmental conditions affecting algal growth, annual variability, and differences in the seasonal timing of sampling. However, we caution that there is also a big difference in the precision and accuracy of evaluating cell numbers and biovolumes using FlowCam where hundreds to several 10ns of thousands of cells are analysed (current study and Chevrollier et al., 2022) compared to the low cell numbers counted using a hemocytometer.

### GIA metabolome indicates adaptation to low phosphorous and low temperature conditions

Here, we have, for the first time, evaluated the endometabolome profiles of surface microbial communities on the GrIS, that have a significant proportion of GIA biomass. We found a total of 804 metabolites (level 2 annotation) with an annotation rate of 20% in GC–MS and 7% in LC–MS/MS analysis The fact that only a small proportion of detected features can be annotated is a common concern in (environmental) metabolomics studies, due to complex chromatograms, peak picking issues, artefacts introduced by chromatographic deconvolution, incomprehensive databases and the ongoing discovery of novel metabolites from unexplored environments (Bundy et al., 2009; Viant et al., 2017).

We found that the anticipated primary metabolite groups (polar and neutral lipids, amino acids, peptides, carbohydrates) were well represented in the annotated metabolome. A comparison with the lipidomic analysis of *A. alaskana* and *A. nordenskioeldi* dominated samples from the Swiss and Austrian Alps by Procházková et al., 2021*,* reveals a larger proportion of diacylglycerides in our samples, rather than a dominance of triacylglycerides. Interestingly, we found a variety of betaine diacylglycerols (Diacylglyceryl-*N,N,N*-trimethylhomo-serine lipids, DGTS), which made up 8% of the identified metabolome, and were previously not reported in GIA. Betaine lipids are polar glycerolipids that occur in some microalgae species and are presumed to replace the function of phosphatidylcholines as an acyl editing hub and fatty acid source in the biosynthesis of triacylglycerols for energy and carbon storage (Hoffmann & Shachar-Hill, 2023). Previous work on the marine microalgae *Nannochloropsis oceanica* suggested that betaine lipids are essential for survival during phosphorous starvation and at low temperatures (Murakami et al., 2018). Such conditions are akin to the oligotrophic and cryophilic environments in which GIA bloom on the GrIS, where the GIA biomine minerals such as hydroxylapatite (McCutcheon et al., 2021) in order to obtain inorganic phosphorous.

### Photochemical analysis and metabolomics results are influenced by sample processing and show a dynamic physiological response in GIA

Lower levels of *F*_*V*_*/F*_*M*_ are associated with a lower efficiency of non-photochemical quenching and indicate previous stress exposure, particularly pointing toward photoinhibition (Maxwell & Johnson, 2000). Previous studies of GIA on the GrIS found *F*_*V*_*/F*_*M*_ ratios between 0.3 and 0.4 (Doting et al., 2024; Halbach et al., 2022; Williamson et al., 2020). Our results showed significantly higher average *F*_*V*_*/F*_*M*_ ratios of 0.49 ± 0.008 in water bath melted samples and 0.57 ± 0.08 across all samples. We expect that the comparatively higher average *F*_*V*_*/F*_*M*_ ratio found in our study is due to the overcast conditions on the day of sampling, lowering the exposition of the glacier algal community to the generally very high levels of ambient irradiation on the ice sheet surface.

There was no evidence of water bath temperature impacting GIA stress levels, but melting and processing duration were clear distinguishing factors (Fig. 3), with significantly lower stress levels in the samples melted at ambient conditions. The ambient-melted samples were kept in a shaded area until processing and melting took 25 h, so it is likely that this effect was due to photosystem II recovery via non-photochemical quenching pathways in the time elapsed. A similar effect was reported for incubations of melted GIA samples under 0, 50 and 100% ambient irradiation by Williamson et al. (2020), who found lower stress levels in shaded samples compared to full irradiation exposure. Our results suggest that *F*_*V*_/*F*_*M*_ ratios level out if the PAM analysis is delayed, because no significant increase was observed 29 h after initial sampling (Fig. 3).

Previous studies of snow and ice microbial endometabolomes (Davey et al., 2019; Fujii et al., 2010; Lutz et al., 2015; Procházková et al., 2021) or targeted ice microbial pigment analysis (Halbach et al., 2022; Williamson et al., 2020) melted samples slowly, either at room temperature (Lutz et al., 2015), under ambient conditions in the field (Williamson et al., 2020), or at controlled cold temperatures (4–5 °C, (Davey et al., 2019; Fujii et al., 2010; Procházková et al., 2021; Remias et al., 2005)) and either in light (Davey et al., 2019) or dark conditions (Halbach et al., 2022; Procházková et al., 2021; Williamson et al., 2020). Melt duration, where reported, was 6–24 h (Halbach et al., 2022; Lutz et al., 2015; Procházková et al., 2021; Williamson et al., 2020), but exact values for melt duration as well as temperature and light conditions are often not provided. Melting at low temperatures constitutes a significant delay to quenching and preservation of GIA metabolomes after removal from their natural habitat, as documented above for ambient melting (Fig. 2). This poses a risk for artefact formation through retained metabolic activity and adaptation to the changed environmental conditions. An alternative method was explored by Lutz et al. (2024) who quenched samples directly by adding methanol to a still frozen sample, to achieve a final concentration of approximately 50% methanol. This evades the long duration until quenching, but methanol addition will also partially lyse the cells, thereby mixing the endometabolome and exometabolome (Kapoore & Vaidyanathan, 2018), which impacts reproducibility, normalization and comparability of samples. Fast sample melting in a water bath has not been reported so far for GIA endometabolome analysis. Water bath melting significantly reduces delays in quenching and sample preservation (here by almost 24 h) and ensures consistent processing conditions. However, the issue persists that the ground truth of the sample metabolome remains unknown as water bath melting still constitutes a delay in sample processing and an alteration of environmental conditions.

In our study, the largest effects on the metabolome and lipidome in our melt procedures, as well as PAM results, were seen in a comparison of AM to WB melted samples (Figs. 3, 5 and 6). Associated effects of temperature and light have not been studied on GIA elsewhere to date, but an effect of culturing temperature on metabolome composition has been reported for an Antarctic *Chlamydomonas sp.* green alga, where soluble sugars, antioxidants and polyamines were increased under cold-stress (Cvetkovska et al. 2022). The distinction between WB and AM seen in the PAM data (Fig. 3) also supports a photophysiological component in the metabolic adaptation post-sampling, likely influenced by the shading of samples during ambient melt.

The 63 differentially affected metabolites include some that have been previously associated with environmental stressors in other microalgae or related *Zygnematophycea* species (Guo et al., 2015; Hoffmann & Shachar-Hill, 2023; Mimouni et al., 2018; Murakami et al., 2018). Glycerolipids and glycerophospholipids are essential membrane structural components and relevant for the physical modulation and adaptation of cell membranes (Mimouni et al., 2018; Stéphanie et al., 2022). The function and modulation of betaine lipids in response to phosphorous starvation and cold stress has been discussed above (Hoffmann & Shachar-Hill, 2023; Murakami et al., 2018). We generally found a reduction of affected betaine lipids in AM samples, indicating a similar role in GIA.

PMeOH in our lipid analysis may stem from PCs via transphosphatidylation due to the use of methanol in quenching and extraction (Tsugawa et al., 2019). However, the intensity of PMeOH lipids directly opposes the effects found for PCs. Therefore, an endogenous production of PMeOH or another convoluting effect is also likely (Fig. 6).

Gallic acid, while cytotoxic at high concentration due to the generation of reactive oxygen species (Guo et al., 2015), is also an important precursor for the photoprotective pigment purpurogallin, that characterizes GIA (Remias et al., 2012a, 2012b; Sang et al., 2004). The decrease of gallic acid in AM samples (Fig. 6) may indicate a photophysiological adaptation in lowering the production of purpurogallin following reduced light exposure for an extended duration. However, this could not be confirmed as our analytical approach (MSTFA derivatization and GC–MS for polar extract) does not cover purpurogallin. Further experiments under controlled light conditions paired with a suitable LC–MS approach (e.g. Remias et al., 2012a, 2012b) to quantify purpurogallin and derivatives are necessary to confirm this effect.

## Conclusions

We assessed the impact of melting strategies on the endometabolome of GIA dominated samples. Our results revealed that 8% of metabolites were affected when samples were allowed to melt uncontrolled at ambient in-field temperature and light conditions compared to controlled fast melt, which showed consistent results at all temperatures. While assessing the GIA metabolome in a truly unbiased manner remains technically challenging, we recommend that future metabolomics studies of supraglacial microbial communities use water bath melting to achieve reproducibility and allow comparisons between studies. The overlapping results for all water bath melt temperatures leads us to recommend melting at 10 °C, as low water bath temperatures are easier to maintain under cold field conditions and closer to the natural field conditions in glacial environments. When longer melting strategies are employed, our data show that the affected metabolites may be a consequence of in situ physiological adaptation of GIA to light during melting. Thus, diurnal and seasonal metabolomics in GIA need to be further investigated. Beyond metabolomic analysis, an effect of melting conditions on GIA photophysiology was also observed in PAM analysis. It is reasonable to assume, that sample processing will affect multiple levels of molecular regulation and adaptation in glacier microbial communities and similar effects will likely be observed in transcriptomic and proteomic analyses of GIA rich samples. Overall, our results highlight the need to take into consideration possible effects of sample processing and preservation in all analyses of mobile molecular elements and to use consistent protocols across the field of snow and ice microbial research.

## Supplementary Information

Below is the link to the electronic supplementary material.

Supplementary file1 (DOCX 4462 KB)

Supplementary file2 (XLSX 77 KB)

Supplementary file3 (CSV 252 KB)

Supplementary file4 (HTML 234 KB)
