# Supplementary material for: Endometabolic profiling of pigmented glacier ice algae: the impact of sample processing
Source: Metabolomics. 2024 Aug 9;20(5):98. doi: 10.1007/s11306-024-02147-6 (PMC11315761; doi:10.1007/s11306-024-02147-6)
Supplement: Supplementary file 1 — Supplementary file1 (DOCX 4462 KB) [file 11306_2024_2147_MOESM1_ESM.docx]

Supplementary Data

Endometabolic profiling of pigmented glacier ice algae – the impact of sample processing

Elisa K. Peter^1,2*^, Carsten Jaeger^3^, Jan Lisec^3^, R. Sven Peters^1^, Rey Mourot^1,2^, Pamela E. Rossel^1^, Martyn Tranter^4^, Alexandre M. Anesio^4^, Liane G. Benning^1,2^

^1^ German Research Centre for Geosciences – GFZ, 14473 Potsdam, Germany

^2^ Department of Earth Sciences, Freie Universität Berlin, 12249 Berlin, Germany

^3^ Bundesanstalt für Materialforschung und -Prüfung, 12489 Berlin, Germany

^4^ Department of Environmental Science, Aarhus University, 4000 Roskilde, Denmark

* Correspondence: Elisa K. Peter [elisa.peter@gfz-potsdam.de](mailto:elisa.peter@gfz-potsdam.de)

# Metabolomics extraction protocol

We extracted GIA samples for metabolomics analysis using a combination of ball milling and freeze-thaw cycles to achieve cell lysis followed by a sequential biphasic solvent extraction based on Sostare et al. (2018) which was optimized for metabolite recovery from GIA. In addition to the protocol provided in the main manuscript a detailed protocol for adaption is provided in the following.

To limit degradation of light and oxidation sensitive metabolites (primarily carotenoids) all work was performed with dimmed lights and on ice and sample tubes were filled with N_2_ prior to storage of dried extracts. All solvents used were UPLC-grade.

|  | Preparation of initial extraction solvent  In a 100 ml Schott Bottle combine:   - 16 ml methanol - CIL Metabolomics QC Mix 1 reconstituted in 1 ml of 50 % MeOH - CIL Metabolomics QC Mix 2 reconstituted in 1 ml of 50 % MeOH - 57 ml MTBE - 75 mg BHT   CIL standards are reconstituted by adding each 500 µl of MilliQ H_2_O and MeOH each to the vial, vortex mixing and ultrasonication. Subsequently pipette CIL mix into the extraction mix and rinse CIL standard vials several times with the extraction mix. The final concentration of internal standards will be 0.06 µg/mL. Mix and pre-cool the solution at -20 °C. |
| --- | --- |
|  | Add two MeOH-cleaned stainless steel milling balls to each sample tube |
|  | Add 1 ml extraction solvent at-15 °C, vortex |
|  | Flash freeze sample tubes in liquid nitrogen for 10 s, followed by 2 min milling in the pre-cooled (-20 °C) sample holder at 30 Hz (MM400, Retsch).  Complete with a final cycle of freeze-thawing followed by 20 min milling. |
|  | Repeat flash freezing and milling twice, with the last milling extended to 10 min. |
|  | Ultrasonicate samples ice-cooled for 15 min (S40 H, Elmasonic) |
|  | Add 500 µl of 25 % MeOH and shake in ball mill for 2 min at 30 Hz to assist phase exchange |
|  | Incubate for 10 min on ice and vortex in between |
|  | Centrifuge (5 min, 14.500 rpm, miniSpin plus, Eppendorf) to assist phase separation and sediment cell debris |
|  | Transfer 700 µl of the upper, organic phase to a fresh 2 mL microcentrifuge tube without disturbing the phase interface |
|  | Add 500 µl MTBE to re-extract |
|  | Shake in ball mill for 2 min |
|  | Incubate on ice for 10 min and vortex in between |
|  | Centrifuge (5 min, 14.500 rpm) |
|  | Transfer 600 µl of the upper organic phase to the microcentrifuge containing the previously removed MTBE extract |
|  | Add 500 µl MTBE to re-extract and repeat steps 12-15 |
|  | Transfer 300 µl of the polar extract (lower phase) into a 15 ml centrifuge tube, without disturbing the sample pellet. The extract is very dark at this stage which is why only a low volume is removed in the first step to avoid disturbing the pellet in low visibility. If the sample pellet is disturbed in the process, re-centrifuge before taking the polar phase. |
|  | Add 800 µl 25 % MeOH to re-extract |
|  | Ultrasonicate ice-cooled for 10 min |
|  | Centrifuge (5 min, 14.500 rpm) |
|  | Transfer 800 µl of the polar extract into the same falcon tube combining with the previous polar extract |
|  | Add 800 µl MilliQ water to re-extract |
|  | Vortex (30s), incubate on ice for 10 min, vortex in between if sample settles |
|  | Centrifuge (5 min, 14.500 rpm) |
|  | Transfer 800 µl of the polar extract into the same falcon tube combining with the previous polar extract |
|  | Re-extract twice more with 800 µl MilliQ water following steps 22-25 |
|  | Centrifuge polar and non-polar extracts (5 min, 14.500 rpm) |
|  | Transfer 1 ml aliquots of the polar extract and 600 µl aliquots of the MTBE extract into 1.5 ml microcentrifuge tubes, dry (30 °C, 1200 rpm, 8 mbar, RVC 2-25 CDplus, Christ), fill headspace with nitrogen gas and store at -80 °C until analysis. |

# Cell counts and morphotype classification via FlowCam analysis

Cell numbers were determined using a FlowCam 5000 particle analyser (Yokowaga Fluid Imaging Technologies) equipped with a 10x objective, using the associated Visual Spreadsheet 5 software. Samples were run at a flow rate of 250 µl/min. The acquired particle images were captured using a pixel threshold of 15 for both white and dark pixels, nearest neighbor distance of 0.1 µm, and close hole iterations of 5. Particles were classified as filamentous and unicellular GIA, as well as snow algae, based on libraries containing 15-35 reference images of particles, originating from the analyzed sample run. Automated particle classification was complemented by manual inspection of images. Figure 1 shows representative images of particle classes, covering the range of appearance of GIA and snow algae. Value filters for geodesic length, geodesic thickness, and geodesic aspect ratio were applied to bin filamentous GIA.

To calculate the number of individual GIA cells in filaments, first an average of GIA cell length was obtained by measuring the length and width of 250-500 individual filamentous GIA cells per sample run in ImageJ. The geodesic length values of full GIA filaments obtained from Visual Spreadsheet were then divided by the average length of individual GIA cells to calculate the total number of cells in filaments. We used the approach of Williamson et al. (2018) for cell volume calculation of GIA based on a cylindrical model (Hillebrand et al. 1999), using cell diameter and cell height measurements obtained in ImageJ on GIA FlowCam images. Carbon content was then calculated using the cell volume to carbon content ratio of Montagnes et al. (1994).


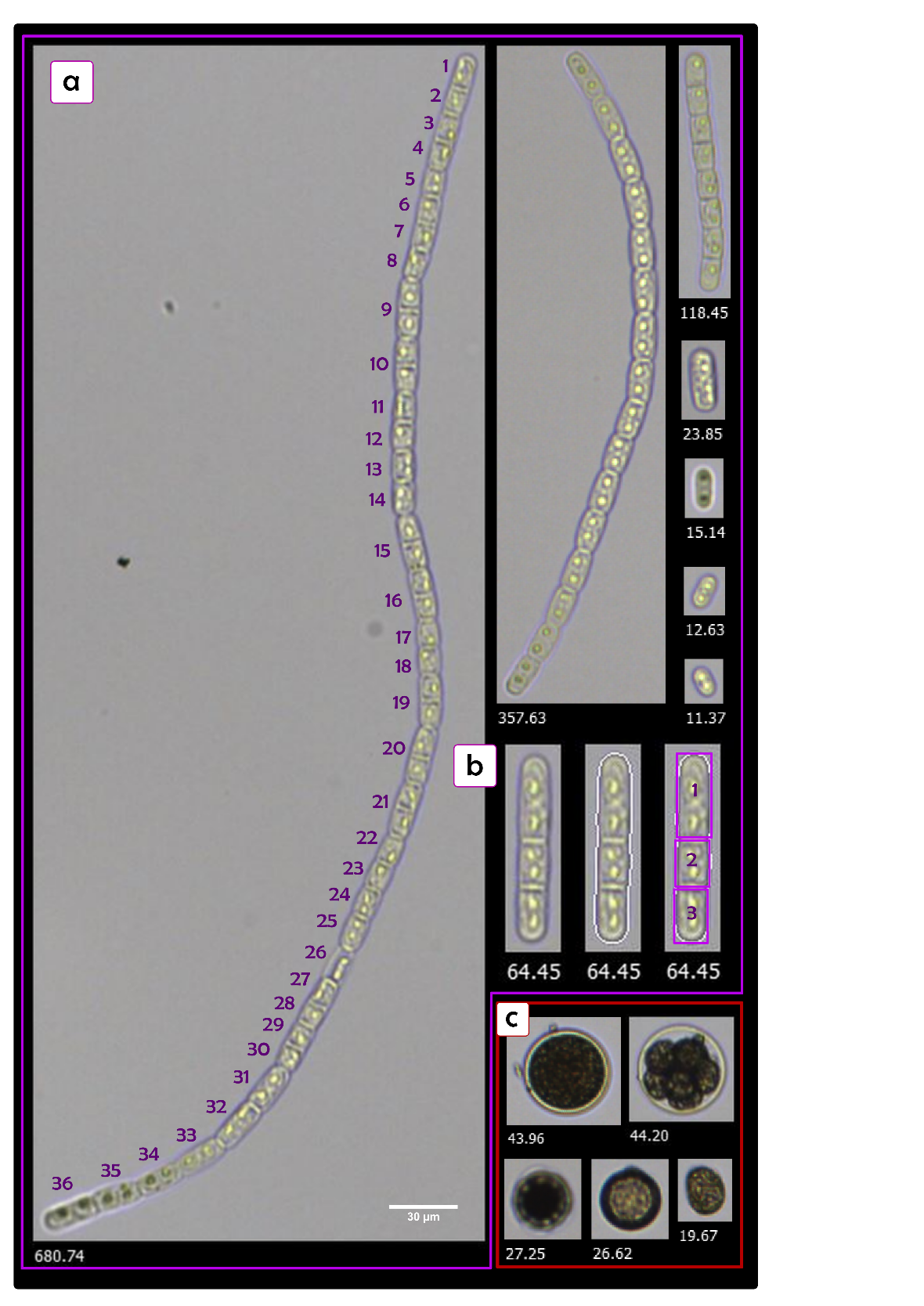


Figure 1: Particle classification from FlowCam analysis. White numbers below images correspond with geodetic length in µm. Purple numbers indicate individually counted cells in filaments. a) unicellular and filamentous GIA, b) 3-celled-filament with clear partitioning wall and potential plastids marking individual cells, the white outline applied by Visual Spreadsheet in the middle picture shows good image focus, c) snow algae cells

Table 1: FlowCam analysis results and sample dry weight for metabolomics extraction

| **Sample** | **Extracted dry weight, metabolomics [mg]** | **GIA in filaments [mL^-1^]** | **Unicellular GIA  [mL^-1^]** | **Snow algae  [mL^-1^]** | **GIA cell volume [µm^3^]** | **GIA Biomass [µgC/mL]** |
| --- | --- | --- | --- | --- | --- | --- |
| **WB10C-1** | 45 | 36468 | 40 | 130 | 2096 | 8 |
| **WB10C-2** | 26 | 23870 | 26 | 80 | 2316 | 6 |
| **WB10C-3** | 31 | 24827 | 17 | 114 | 2070 | 5 |
| **WB15C-1** | 31 | 48648 | 54 | 195 | 2116 | 11 |
| **WB15C-2** | 36 | 37338 | 57 | 153 | 2313 | 9 |
| **WB15C-3** | 42 | 32350 | 90 | 88 | 1882 | 6 |
| **WB15C-4** | 35 | 53793 | 61 | 170 | 1935 | 11 |
| **WB20C-1** | 29 | 52087 | 70 | 199 | 2464 | 13 |
| **WB20C-2** | 25 | 56011 | 44 | 144 | 2124 | 12 |
| **WB20C-3** | 39 | 36137 | 26 | 127 | 1975 | 7 |
| **WB20C-4** | 25 | 32563 | 20 | 70 | 2046 | 7 |
| **AM25h-1** | 38 | 45827 | 63 | 328 | 2333 | 11 |
| **AM25h-2** | 32 | 38013 | 45 | 144 | 2245 | 9 |
| **AM25h-3** | 41 | 42807 | 41 | 165 | 1912 | 9 |
| **AM25h-4** | 44 | 42470 | 55 | 162 | 1829 | 8 |
| **AM29h-1** | 37 | 33678 | 87 | 214 | 2402 | 8 |
| **AM29h-2** | 32 | 22219 | 57 | 67 | 1958 | 5 |
| **AM29h-3** | 32 | 37345 | 62 | 125 | 2045 | 8 |
| **AM29h-4** | 35 | 30679 | 47 | 101 | 2530 | 8 |
| **AM42h-1** | 28 | 38411 | 68 | 164 | 2121 | 8 |
| **AM42h-2** | 29 | 25109 | 29 | 47 | 1737 | 5 |
| **AM42h-3** | 86 | 28068 | 52 | 95 | 2016 | 6 |
| **AM42h-4** | 63 | 57538 | 64 | 172 | 2265 | 14 |
| **AM49h-1** | 38 | 35886 | 28 | 64 | 2432 | 9 |
| **AM49h-2** | 21 | 28115 | 28 | 44 | 2075 | 6 |
| **AM49h-3** | 53 | 47412 | 58 | 98 | 2146 | 11 |
| **Average** | 37 | 37987±9981 | 51±19 | 133±61 | 2130±202 | 8±0.2 |

# Microbial diversity

Following DNA extraction, DNA concentration in the extract was measured on a Qubit 3.0 (Invitrogen, Eugene, Oregon, US) with the broad-range dsDNA kit (Invitrogen). Extracted DNA was amplified with prokaryotic primers for the 16S rRNA gene Bakt_341F (CCTACGGGNGGCWGCAG) and Bakt_805R (GACTACHVGGGTATCTAATCC) (Herlemann et al. 2011), eukaryotic primers for the 18S rRNA gene using 528F (5’- GCGGTAATTCCAGCTCCAA-3‘) and 706R (5’-AATCCRAGAATTTCACCTCT-3‘) (Cheung et al. 2010), and the internal transcribed spacer 2 (ITS2 snow) gene primers 5.8SbF (5′-GATGAAGAACGCAGCG-3′; (Mikhailyuk et al. 2008)) and ITS4R (5′-TCCTCCGCTTATTGATATGC-3′; (White et al. 1990)) and the ITS2 ice primers 5.8SbF (5’ - CGATGAAGAACGCAGCG-3’) and LSULP (5’ – AATTCGGCGGGTGGTCTTG – 3’) (Remias et al. 2023). The first PCRs contained 12.5 μL of 2x PCRBIO Ultra Mix (PCR Biosystems), 0.5 μL of forward and reverse primer from a 10 μM stock, 0.5 μL of bovine serum albumin (BSA) to a final concentration of 0.025 mg/mL, 0.6 μL of sterile water and 5 μL of template. Conditions were as follows: at 95 ˚C for 2 min, followed by 38 cycles of 95 ˚C for 15 sec, 55 ˚C for 15 sec, 72 ˚C for 40 sec, with a final extension performed at 72 ˚C for 4 min. An electrophoresis 1% agarose gel was run with all the PCR products before proceeding to the second PCR. PCR bands were purified from the gel, and samples were subsequently indexed in a second PCR. 5 µl of product from the purified PCR was used as template to add indexes and sequencing adaptors in a reaction consisting of 12.5 μl of 2x PCRBIO Ultra Mix (PCR Biosystems), 2 μl of each index primer (P5/P7), and DNase free water to a final volume of 28 µl. For the second PCR, conditions were as follows, pre-incubation at 98 °C for 1 min, followed by 13 cycles of 98 °C for 10 sec, 55 °C for 20 sec, and 72 °C for 40 sec, and ending with a final step at 72 °C for 5 min. The final PCR products were purified with 15 µl HighPrep PCR magnetic beads (MagBio Genomics Inc. Gaithersburg, Maryland, US) according to the manufacturer's instructions and eluted in 27 µl TE buffer. Aliquots of the PCR products were run on a 1.5% agarose gel and checked under UV light. Concentrations of the amplified and purified DNA samples were re-measured on the Qubit 2.0 fluorometer (Invitrogen, Eugene, Oregon, US). Subsequently, the sample was equimolarly pooled, and this final library was sequenced on an Illumina MiSeq using the V2 kit (Illumina Inc. SanDiego, California, US) resulting in 2×250 bp reads. Extraction, amplification and sequencing controls have been performed at each step to ensure the reliability of the sample treatment and control for potential contamination.

The raw sequencing data from all four targeted marker genes (16S rRNA, 18S rRNA, ITS2 snow rRNA and ITS2 ice rRNA) were pre-processed using the DADA2 (vers 1.16) R (4.2.2) package (Callahan et al. 2016) for primer, non-target length sequences and chimera removal, and reads merging. Sequences tables were merged and taxonomy was assigned for 16S and 18S rRNA gene sequences using the SILVA v138 database, while ITS2 snow and ITS2 ice gene sequences were assigned using the UNITE database (Nilsson et al. 2019, version 9). The dominant 18S ASVs which were not assigned at the Order level at this step were blasted by hand. Taxa which were not assigned at the genus level were marked as belonging to the upper taxonomic rank to be considered using the function tax_fix() from the package microViz (Barnett et al. 2021) (0.10.8). An additional filtering step was performed to remove singletons before the analysis. Data was assembled and processed under the form of a Phyloseq (McMurdie and Holmes 2013) (vers. 1.42.0) object. After control of our dataset, blanks were excluded. Taxonomic ranks which were left non-assigned were assigned to the superior known taxonomic rank using the tax_fix() function (Barnett et al. 2021).


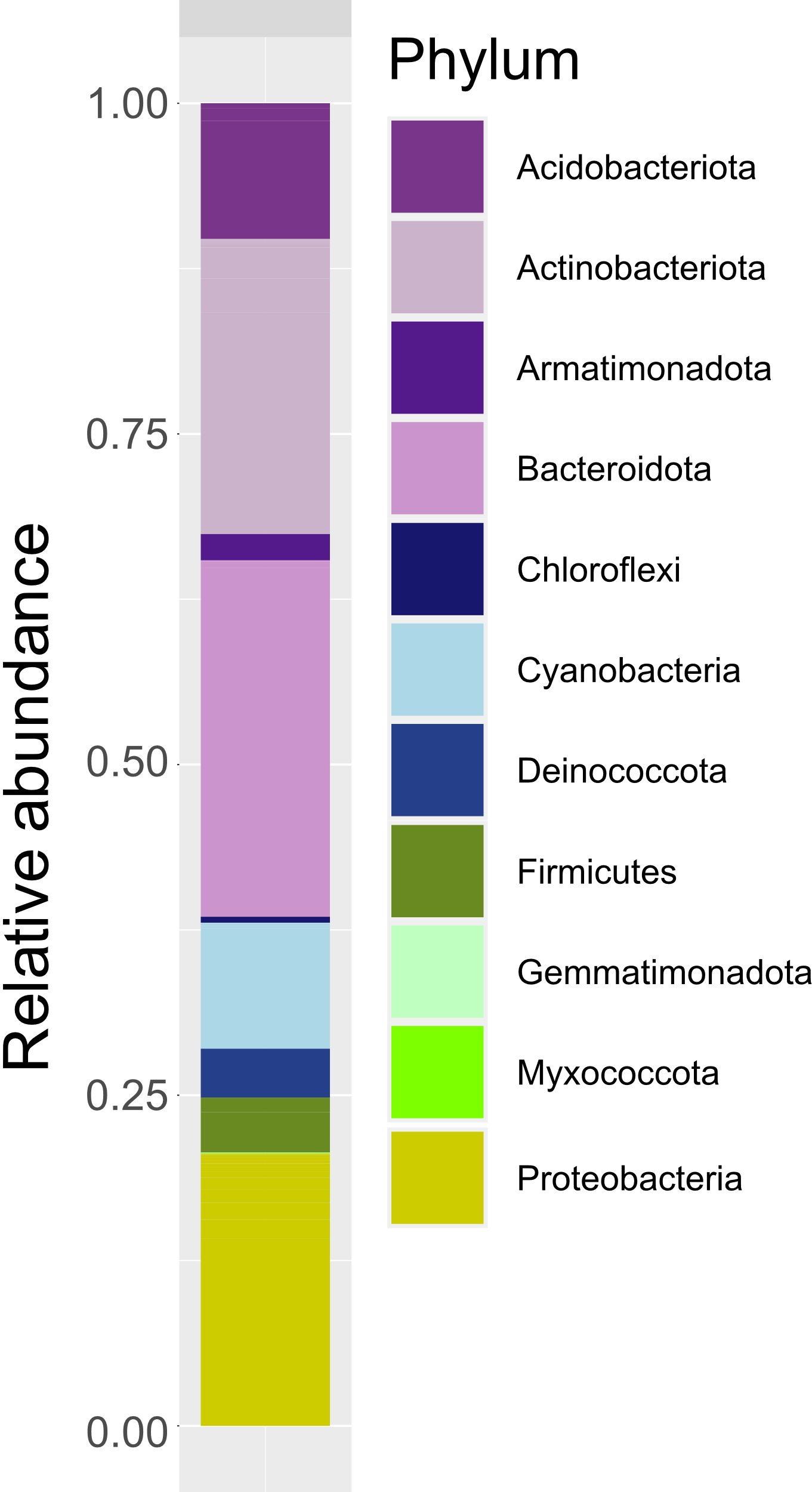

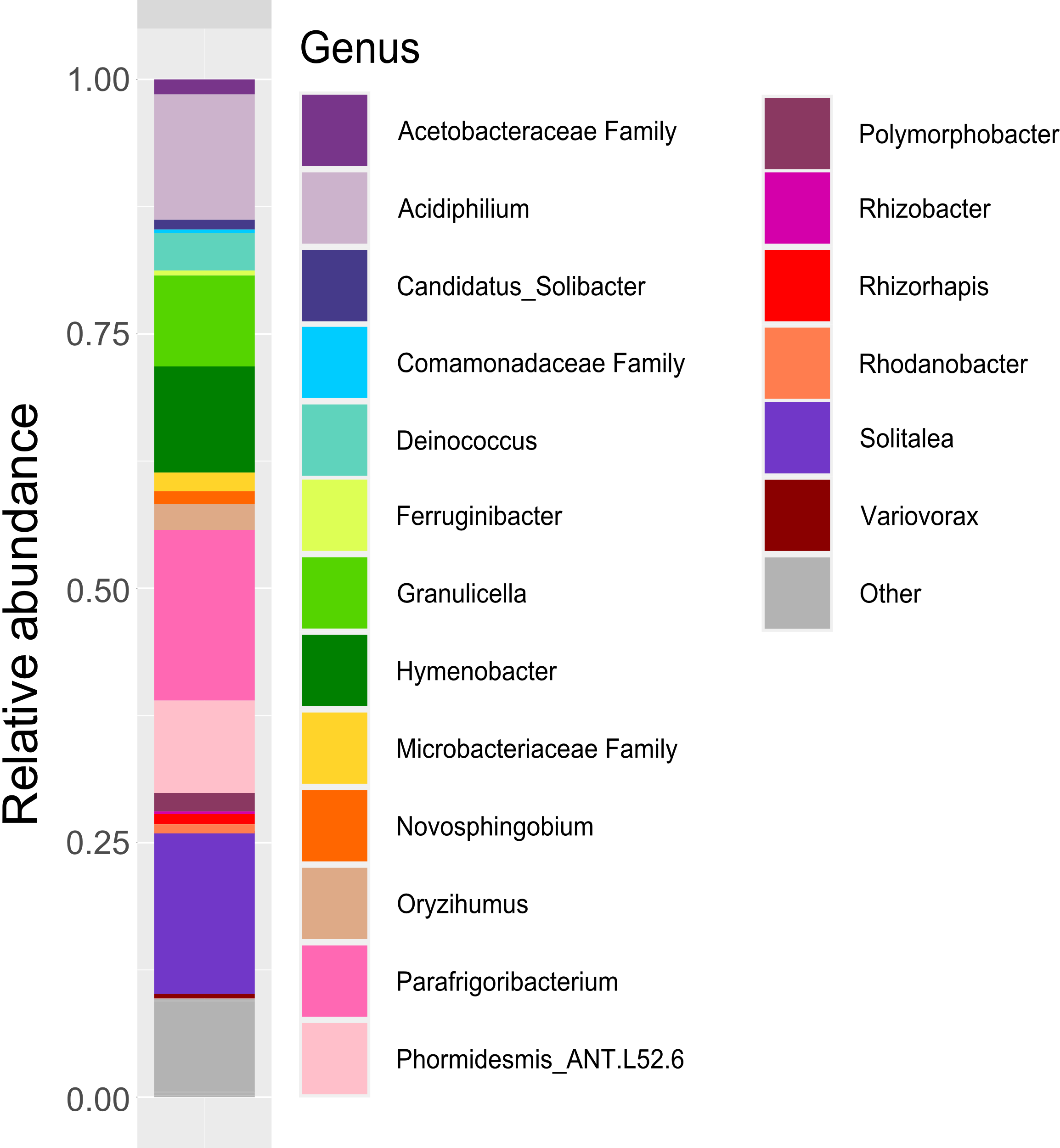


Figure 3: Relative abundance plots showing the prokaryotic microbial community composition based on 16S DNA amplicon sequencing by phylum (left) and genus (right).


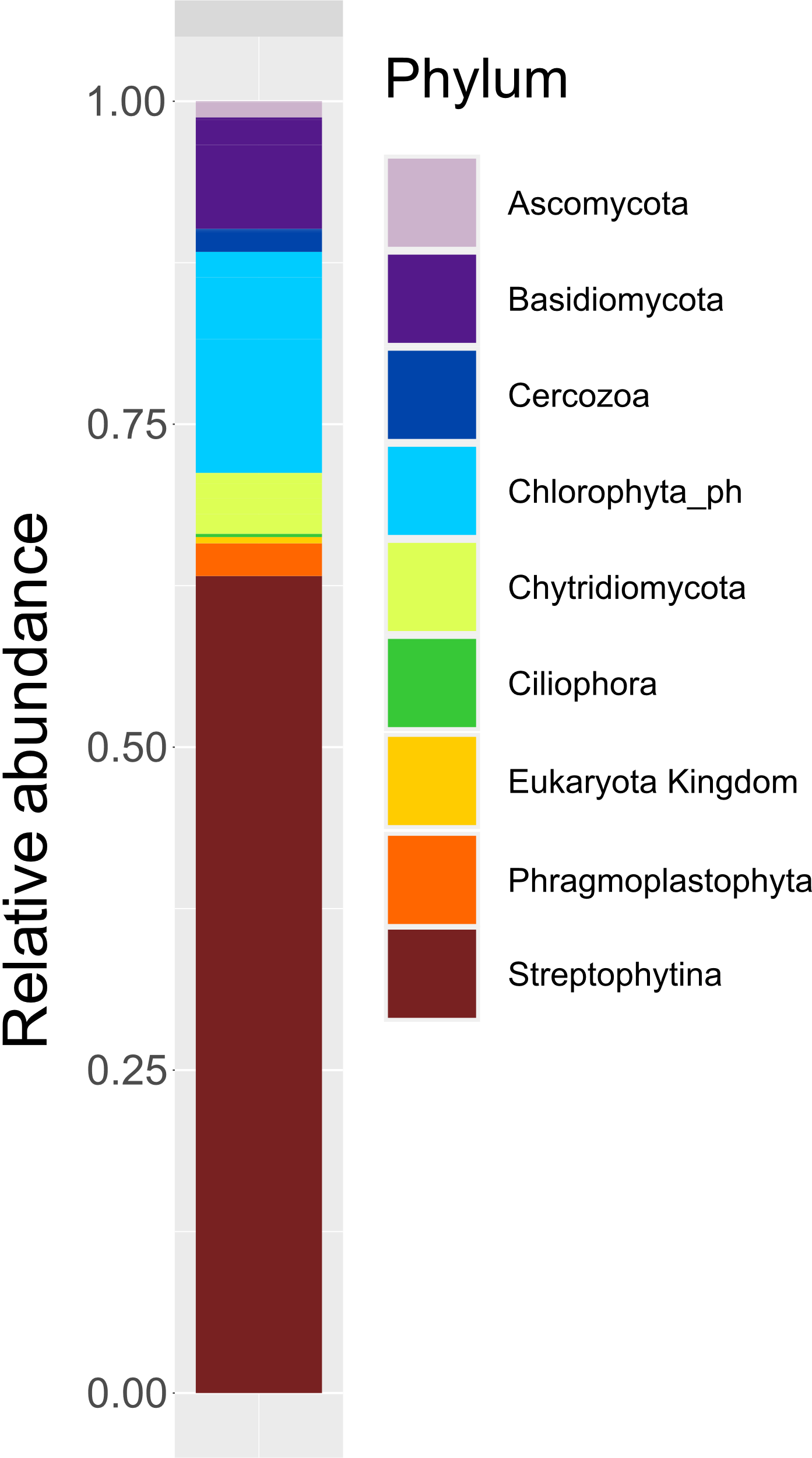

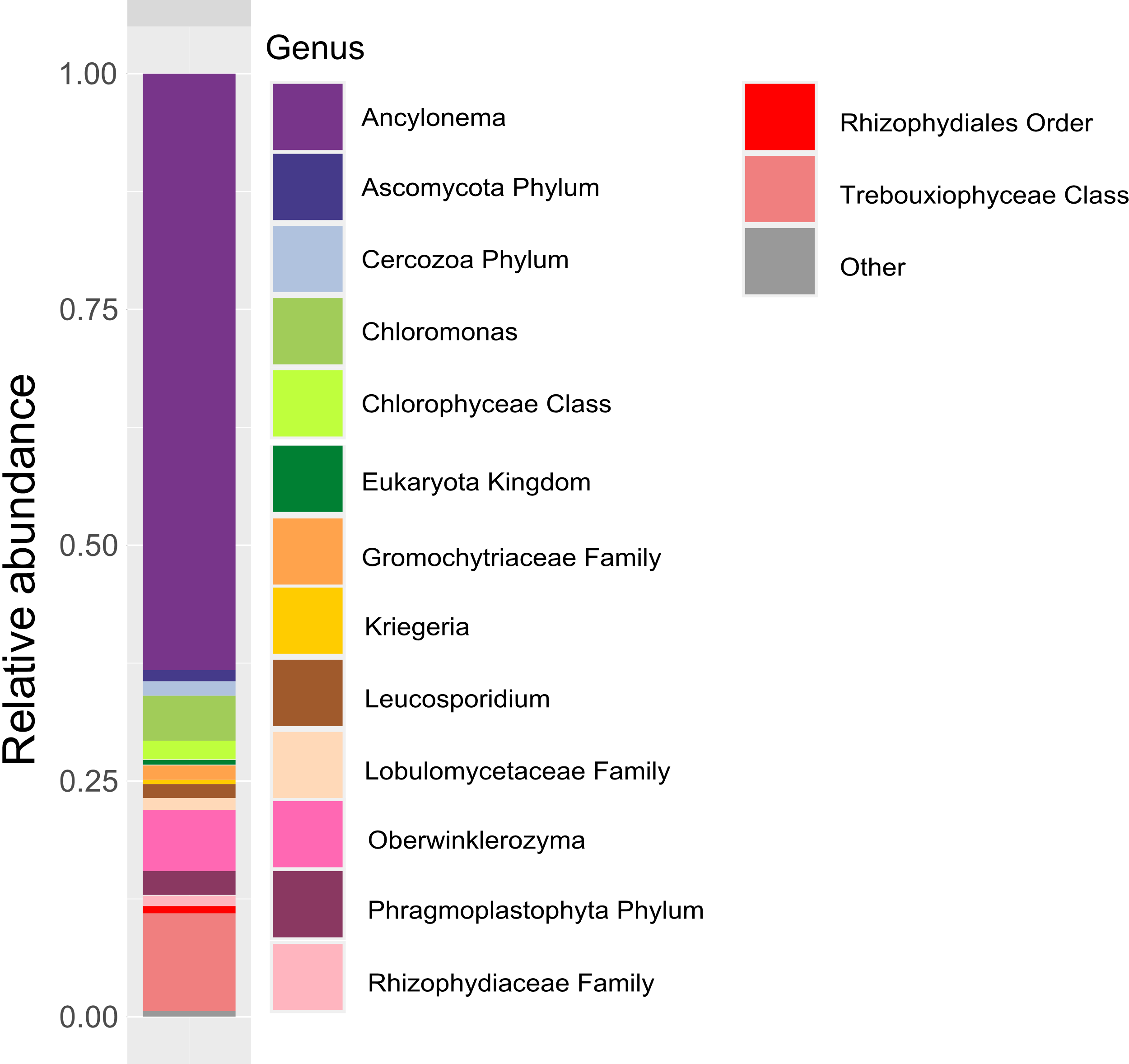


Figure 4: Relative abundance plots showing the eukaryotic microbial community composition based on 18S DNA amplicon sequencing by phylum (left) and genus (right).


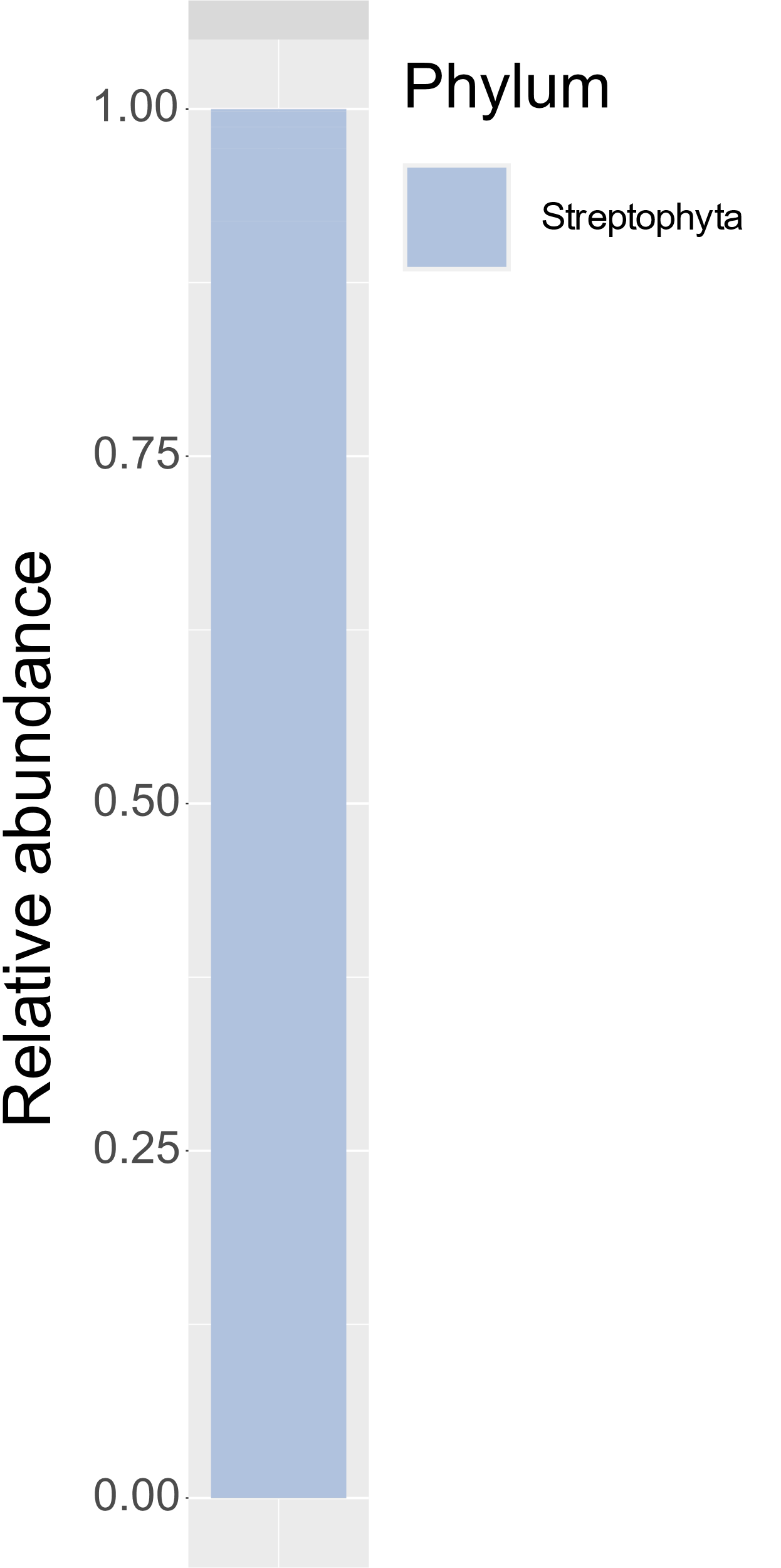

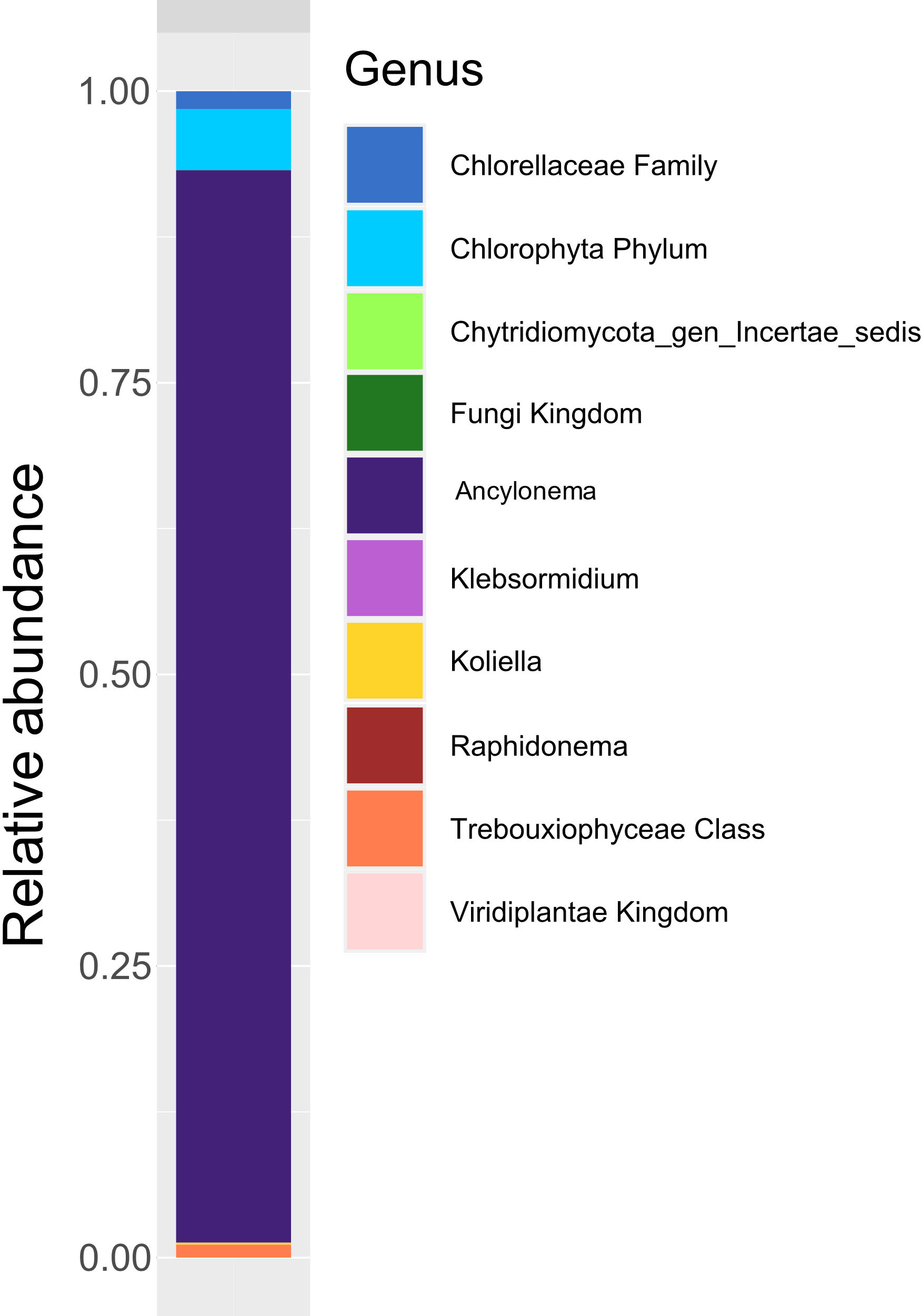


Figure 5: Relative abundance plots showing the fungal microbial community composition based on ITS-2 ice DNA amplicon sequencing by phylum (left) and genus (right).


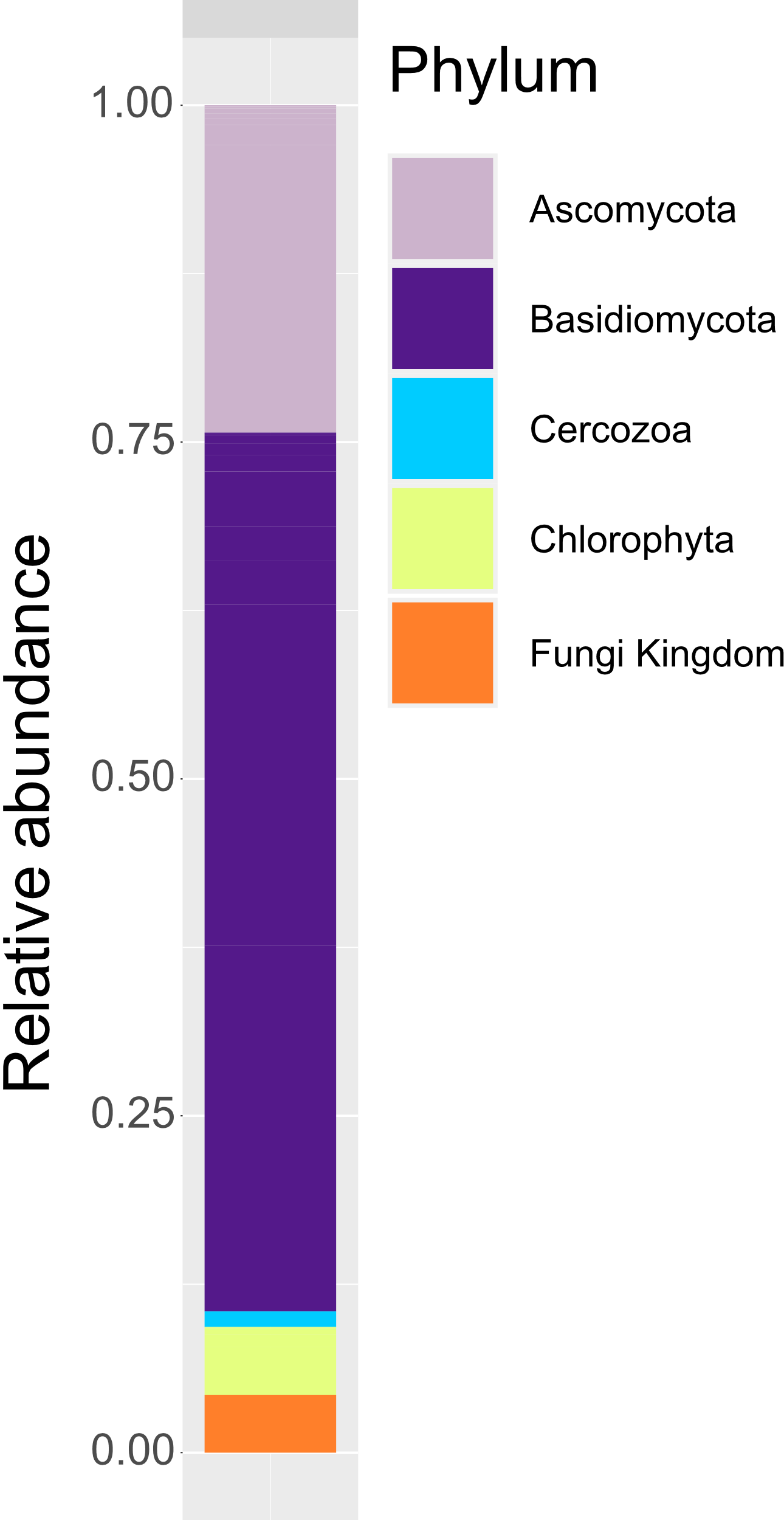

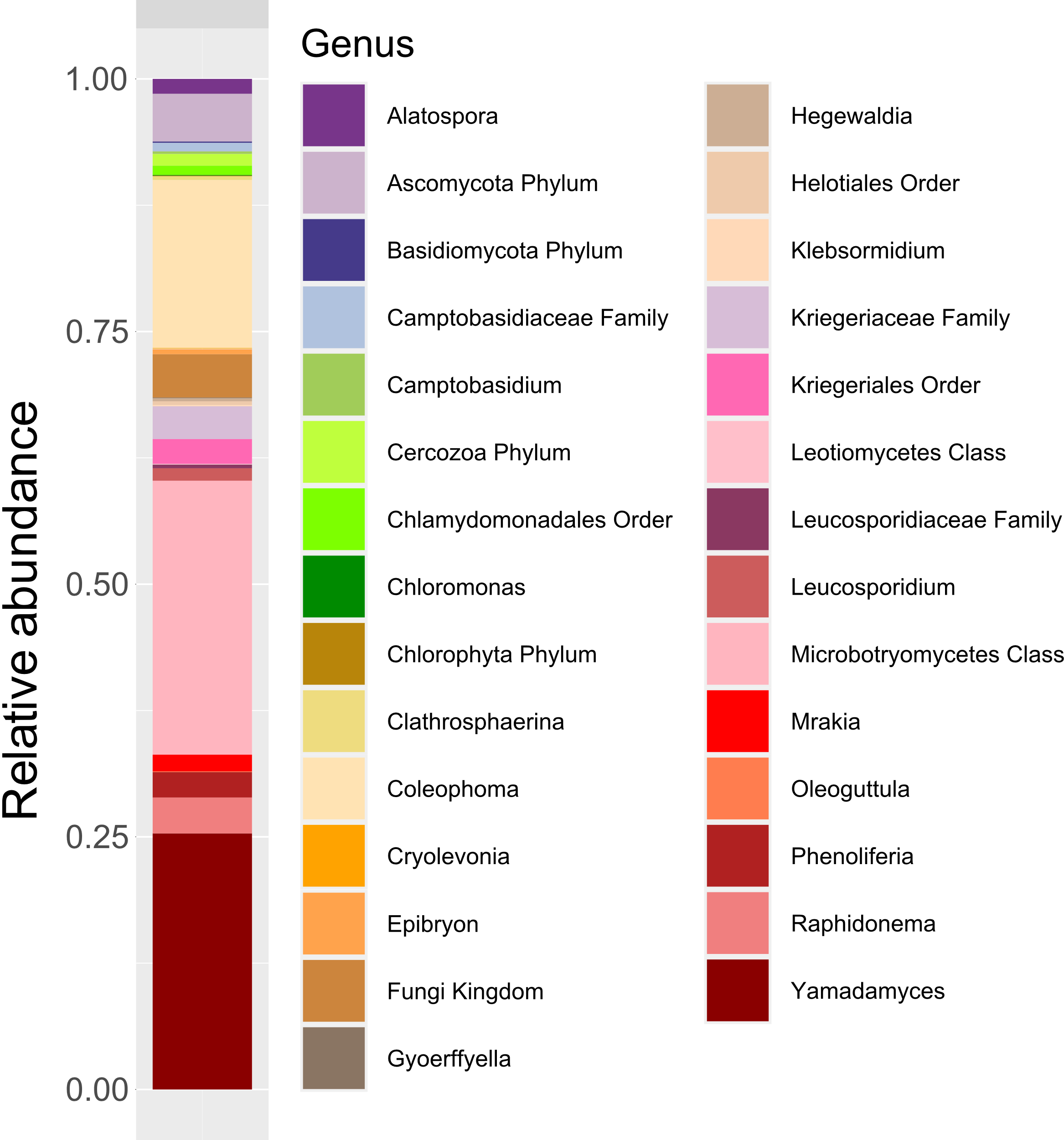


Figure 6: Relative abundance plots showing the fungal microbial community composition based on ITS-2 snow DNA amplicon sequencing by phylum (left) and genus (right).

# References

Barnett, D. J. M., Arts, I. C. W., & Penders, J. (2021, June 30). microViz: an R package for microbiome data visualization and statistics. Zenodo. https://doi.org/10.5281/zenodo.5048013

Callahan, B. J., McMurdie, P. J., Rosen, M. J., Han, A. W., Johnson, A. J. A., & Holmes, S. P. (2016). DADA2: High-resolution sample inference from Illumina amplicon data. *Nature Methods*, *13*(7), 581–583. https://doi.org/10.1038/nmeth.3869

Cheung, M. K., Au, C. H., Chu, K. H., Kwan, H. S., & Wong, C. K. (2010). Composition and genetic diversity of picoeukaryotes in subtropical coastal waters as revealed by 454 pyrosequencing. *The ISME Journal*, *4*(8), 1053–1059. https://doi.org/10.1038/ismej.2010.26

Herlemann, D. P., Labrenz, M., Jürgens, K., Bertilsson, S., Waniek, J. J., & Andersson, A. F. (2011). Transitions in bacterial communities along the 2000 km salinity gradient of the Baltic Sea. *The ISME Journal*, *5*(10), 1571–1579. https://doi.org/10.1038/ismej.2011.41

Hillebrand, H., Dürselen, C.-D., Kirschtel, D., Pollingher, U., & Zohary, T. (1999). Biovolume Calculation for Pelagic and Benthic Microalgae. *Journal of Phycology*, *35*(2), 403–424. https://doi.org/10.1046/j.1529-8817.1999.3520403.x

McMurdie, P. J., & Holmes, S. (2013). phyloseq: An R Package for Reproducible Interactive Analysis and Graphics of Microbiome Census Data. *PLoS ONE*, *8*(4), e61217. https://doi.org/10.1371/journal.pone.0061217

Mikhailyuk, T., Sluiman, H., Massalski, A., Mudimu, O., Demchenko, E., Kondratyuk, S. Y., & Friedl, T. (2008). New streptophyte green algae from terrestrial habitats and an assessment of the genus Interfilum (Klebsormidiophyceae, Streptophyta). *Journal of Phycology*, *44*, 1586–1603. https://doi.org/10.1111/j.1529-8817.2008.00606.x

Montagnes, D. J. S., Berges, J. A., Harrison, P. J., & Taylor, F. J. R. (1994). Estimating carbon, nitrogen, protein, and chlorophyll *a* from volume in marine phytoplankton. *Limnology and Oceanography*, *39*(5), 1044–1060. https://doi.org/10.4319/lo.1994.39.5.1044

Nilsson, R. H., Larsson, K.-H., Taylor, A. F. S., Bengtsson-Palme, J., Jeppesen, T. S., Schigel, D., et al. (2019). The UNITE database for molecular identification of fungi: handling dark taxa and parallel taxonomic classifications. *Nucleic Acids Research*, *47*(D1), D259–D264. https://doi.org/10.1093/nar/gky1022

Remias, D., Procházková, L., Nedbalová, L., Benning, L. G., & Lutz, S. (2023). Novel insights in cryptic diversity of snow and glacier ice algae communities combining 18S rRNA gene and ITS2 amplicon sequencing. *FEMS Microbiology Ecology*, fiad134. https://doi.org/10.1093/femsec/fiad134

Sostare, J., Di Guida, R., Kirwan, J., Chalal, K., Palmer, E., Dunn, W. B., & Viant, M. R. (2018). Comparison of modified Matyash method to conventional solvent systems for polar metabolite and lipid extractions. *Analytica Chimica Acta*, *1037*, 301–315. https://doi.org/10.1016/j.aca.2018.03.019

White, T., Bruns, T., Lee, S., Taylor, J., Innis, M., Gelfand, D., & Sninsky, J. (1990). Amplification and Direct Sequencing of Fungal Ribosomal RNA Genes for Phylogenetics. In *Pcr Protocols: a Guide to Methods and Applications,* (Vol. 31, pp. 315–322).

Williamson, C. J., Anesio, A. M., Cook, J., Tedstone, A., Poniecka, E., Holland, A., et al. (2018). Ice algal bloom development on the surface of the Greenland Ice Sheet. *FEMS Microbiology Ecology*, *94*(3), 1–10. https://doi.org/10.1093/femsec/fiy025
