# Supplementary material for: Endometabolic profiling of pigmented glacier ice algae: the impact of sample processing
Source: Metabolomics. 2024 Aug 9;20(5):98. doi: 10.1007/s11306-024-02147-6 (PMC11315761; doi:10.1007/s11306-024-02147-6)
Supplement: Supplementary file 4 — Supplementary file4 (HTML 234 KB) [file 11306_2024_2147_MOESM4_ESM.html]

Javascript must be enabled to view this page.

magnitude

ME

 804

 5

 3

 2

 1

 1

 1

 1

 1

 113

 77

 1

 1

 58

 17

 1

 1

 31

 22

 4

 5

 4

 4

 319

 64

 64

 169

 169

 17

 3

 6

 8

 69

 4

 65

 118

 2

 2

 28

 3

 3

 3

 19

 26

 1

 2

 5

 18

 17

 2

 1

 14

 3

 3

 4

 4

 37

 2

 35

 1

 1

 4

 1

 1

 3

 1

 1

 1

 6

 6

 6

 2

 1

 1

 1

 1

 50

 42

 36

 1

 4

 1

 4

 1

 2

 1

 2

 1

 1

 1

 1

 1

 1

 7

 7

 7

 59

 59

 3

 55

 1

 19

 4

 4

 3

 3

 1

 1

 4

 4

 1

 1

 1

 1

 4

 1

 3

 1

 1

 1

 1

 1

 3

 1

 1

 1

 1

 1

 1

 84

 60

 23

 4

 17

 6

 10

 7

 1

 3

 3

 17

 17

 14

 1

 1

 1

 1

 4

 4

 8

 8
